# Behavioral inflexibility through overtraining is mediated by reduced mGluR1/5 signaling capacity in the dorsolateral striatum

**DOI:** 10.1371/journal.pbio.3003288

**Published:** 2025-07-29

**Authors:** Vincent Paget-Blanc, Anna Cavaccini, Alessandra Longaretti, Luca Nava, Massimo Trusel, Anna Rocchi, Maria Pennuto, Elena Marcello, Fabrizio Gardoni, Barbara Greco, Raffaella Tonini

**Affiliations:** 1 Neuromodulation of Cortical and Subcortical Circuits Laboratory, Istituto Italiano di Tecnologia, Genova, Italy; 2 Neuroscience and Brain Technologies Department, Istituto Italiano di Tecnologia, Genova, Italy; 3 Department of Biomedical Sciences, University of Padova, Padova, Italy; 4 Veneto Institute of Molecular Medicine (VIMM), Padova, Italy; 5 Department of Pharmacological and Biomolecular Sciences, University of Milano, Milano, Italy; Center for Brain Research, Medical University of Vienna, AUSTRIA

## Abstract

The control of instrumental actions engages distinct behavioral strategies whose contributions are regulated with experience. Instrumental performance, which depends on the causal relationship between actions and their outcomes (A–O), relies on flexible, goal-directed control of behavior. Actions can become less sensitive to changes in action–outcome (A–O) contingencies with repetition, resulting in more inflexible, habitual behaviors. The loss of flexibility with repetition requires plasticity at corticostriatal circuits. However, the underlying molecular mechanisms are not yet established, and how these mechanisms specifically relate to the inability to adapt to new contingencies is unknown. In mice, we find that inflexible behavioral performance following overtraining of an appetitive instrumental task is associated with a reduced capacity of mGluR5 receptors in the dorsolateral striatum (DLS) to engage intracellular signaling in response to changes in action–outcome contingency. We also observed dichotomous modulation of timing-dependent synaptic depression (tLTD) at striatal projection neurons of the indirect (iSPNs) and direct (dSPNs) pathways. Preventing overstimulation of mGluR5 signaling through a homotypic process preserved behavioral sensitivity to changes in A–O contingencies despite overtraining, and averted the related biochemical and synaptic changes. Furthermore, mGluR5 couples to different signaling pathways to regulate tLTD in iSPNs and dSPNs. Our findings demonstrate that decreased signaling capacity of mGluR1/5, accompanied by cell-type-specific modulation of corticostriatal synapses in the DLS, represents a key molecular mechanism underlying overtraining-induced behavioral inflexibility.

## Abstract 2

Instrumental actions rely on flexible, goal-directed behavior, but can become more rigid and habit-like with repetition. This study shows that decreased adaptive signaling capacity of striatal mGluR1/5 is key to the loss of behavioral flexibility that accompanies habitual control of behavior.

## Introduction

The ability to adapt behavior (instrumental response) to an ever-changing environment requires actions to be under flexible, goal-directed control. Such control depends on the causal relationship between an action and its outcome (A–O). With repetition, instrumental performance can become habitual, for example when the behavioral contingencies remain constant or outcomes are predictable over time. Habitual behavior is less responsive to changes in A–O associations (i.e., more inflexible) and is primarily elicited by antecedent stimuli [1–3]. The gradual loss of flexibility with repetition is subserved by corticostriatal circuits, which are modulated by the dopamine ascending neuromodulatory system and local neuromodulatory signals (i.e., endocannabinoids) [4–8].

Learning processes associated with goal-directed and habitual actions can be identified during instrumental conditioning [reviewed by [6,9,10]]. The task-related activity of the principal striatal projection neurons (SPNs) of the dorsomedial striatum (DMS) and dorsolateral striatum (DLS) appears to be modulated by experience throughout instrumental learning [4–6,11]. The classical view posits that the DMS mainly supports goal-directed behavior, while the DLS gradually encodes stimulus-response associations through the repetition of behavior, leading to the automation of actions [2,5,12–19]. This view is consistent with habit learning requiring synaptic plasticity at cortical connections to DLS SPNs [20,21]. SPNs functionally segregate into two distinct neuronal populations: the dopamine D1 receptor-expressing SPNs of the direct pathway (dSPNs) and the dopamine D2 receptor-expressing SPNs of the indirect pathway (iSPNs). Habit formation induced by exposure to addictive drugs [20] or task overtraining [8,20,22] specifically affects long-term synaptic depression (LTD) in the iSPNs of the DLS. In contrast, similar synaptic adaptations in DLS dSPNs have not yet been identified, nor have the precise underlying molecular mechanisms.

During prolonged instrumental training, sustained activation of metabotropic receptors involved in synaptic plasticity may progressively limit the ability of SPNs to undergo molecular adaptations in response to changes in action–outcome contingencies, contributing to habitual behavior [23–26]. The Gq/11-coupled metabotropic receptors (mGluR1/5) are ideally present at striatal circuits to be actively recruited during instrumental learning to shape synaptic plasticity [27,28]. At cortico-SPN synapses, mGluR1/5 post-synaptically integrates the cortical glutamate and midbrain dopamine signals critical for encoding A–O associations [29,30], by modulating glutamate release via retrograde endocannabinoid (eCB) signaling [27,31] and the postsynaptic response of SPNs to dopamine via NMDA receptor (NMDAR) regulation [32,33]. Previous studies have pointed to the significance of striatal mGluR5 and NMDAR in memory retention, perseverative behaviors, and extinction learning [34–36]. Nevertheless, it remains unclear whether changes in mGluR5 signaling capacity and associated synaptic plasticity modifications are directly linked to deficits in encoding action–outcome associations following overtraining. In this study, we investigated how adaptations of mGluR5 responsiveness contributes to the cell-type-specific modulation of corticostriatal synapses when mice are challenged with changes in previously learned action–outcome associations during an appetitive instrumental task (i.e., habit training) [8,24]. We specifically focused on the DLS because of the role of this striatal subregion in the development and execution of habitual behaviors [7,14,16,37,38].

## Results

### Instrumental conditioning for food engages mGluR1/5 in the DLS

**Fig 1:**
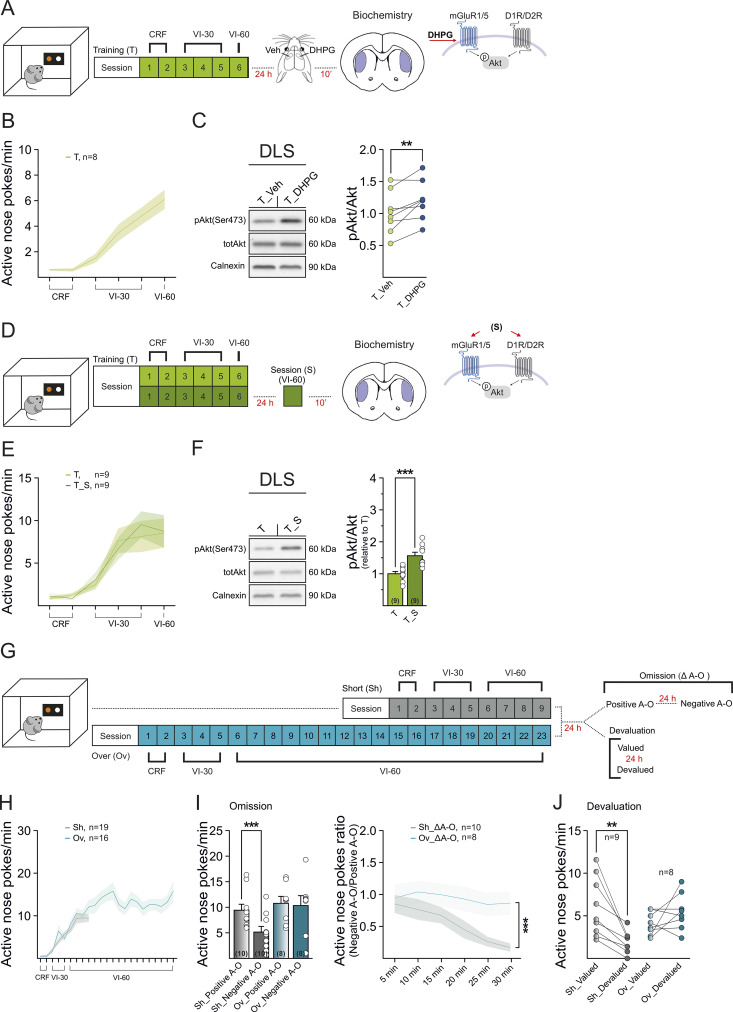
DLS mGluR1/5 signaling activation in instrumental conditioning and differential behavioral sensitivity to A–O changes with training duration. **(A, D)** Schematic of behavioral regimes followed by protein analysis, and targeted intracellular signaling cascades. **(B)** Active nose-poke (ANP) rates (ANP/min) during training of instrumental conditioning for food reward (T_Veh/T_DHPG *n* = 8). **(C)** Representative western blots of pAkt, Akt, and Calnexin (loading control) in response to in vivo infusion of vehicle or DHPG (i.c., 9 ng/0.5 µl) into the DLS. Plots represent quantified data groups of pAkt/Akt (T_Veh_pAkt/Akt_, 1.00 ± 0.12, *n* = 8; T_DHPG_pAkt/Akt_, 1.21 ± 0.11, *n* = 8; T_Veh_pAkt/Akt_ vs*.* T_DHPG_pAkt/Akt_ paired *t* test, **p = 0.01, *t* = 3.41, *dF* = 7). **(E)** ANP rates during training in the two experimental groups (T *n* = 9; T_S *n* = 9). **(F)** Representative western blots of pAkt, Akt, and Calnexin in response to an additional VI-60 session performed 24 h after the end of the training. Bar graphs show expression level ratios (relative to T) of pAkt/Akt (T_pAkt/Akt_: 1.00 ± 0.07, *n* = 9; T_S_pAkt/Ak_: 1.57 ± 0.11, *n* = 9; T_pAkt/Akt_ *vs.* T_S_pAkt/Akt_ unpaired *t* test, ***p = 0.0006, *t* = 4.29, *dF* = 16). **(G)** Schematic of the short and overtraining regimes, followed by post-training procedures. **(H)** ANP rates during instrumental learning in short- (Sh, *n* = 19) and overtrained mice (Ov, *n* = 16). **(I)** Post-training omission procedure. (Left) Comparison of ANP rates between positive and negative A–O contingency in both short- and overtrained mice (Sh *n* = 10, positive A–O: 9.4 ± 1.2, negative A–O: 5.2 ± 1.1, Sidak ***p = 0.001; Ov *n* = 8, positive A–O: 10.8 ± 1.4, negative A–O 10.4 ± 1.9; Sidak *p* = 0.9). (Right) Time courses of ANP ratios (ANP rates under negative A–O/ANP rates under positive A–O) in short- and overtrained mice. **(J)** Post-training devaluation procedure in short- and overtrained mice (Sh *n* = 9, Ov *n* = 8). ANP rates in the valued and devalued conditions (Sh, valued: 6.0 ± 1.1; devalued 2.2 ± 0.5, Sidak **p = 0.001; Ov, valued: 4.1 ± 0.47, devalued: 5.5 ± 0.76, Sidak *p* = 0.32). (**B, E, F, H, I**) Data are presented mean ± standard error of the mean (SEM). Data set are available at the following link: https://doi.org/10.48557/VCAWUD.

As a first step to examine the signaling capacity of mGluR1/5 in the DLS following task overtraining (i.e., habit training), we tested whether mGluR1/5 are engaged during appetitive instrumental conditioning. We initially monitored the Akt pathway, which can act downstream of both mGluR1/5 and dopamine D1 and D2 receptors [39]. First, we used a pharmacological approach to confirm that mGluR1/5 can signal to the Akt-pathway in the DLS at the early training stage. Male P45–P60 mice were trained to nose-poke for a food reward under a variable-interval schedule of reinforcement (Fig 1A), as described [24,40]. We acknowledge that results may be specific to this sex and age range, and may not be directly applicable to females or other developmental stages without further research.

Behaving mice bearing bilateral indwelling cannulae in the DLS, which increase active nose pokes (ANP) throughout training (ANP/min, session: *F*_5,45 _= 41, ****p < 0.0001; Fig 1B), were unilaterally infused with the mGluR1/5 agonist DHPG (9 ng/0.5 μl; T_DHPG) 24 h after the training ended. The contralateral DLS received vehicle solution (T_Veh). Ten minutes after infusion, we assessed the phosphorylation of Akt (pAkt) at Ser473, the site responsible for the activation of Akt signaling [41,42]. Compared to their respective total protein amounts (tot), pAkt protein levels were significantly higher in the DLS injected with DHPG than in the vehicle-infused DLS (pAkt/Akt, **p = 0.01; Fig 1C).

We next tested whether instrumental learning similarly recruited Akt signaling in the DLS via mGluR5 activation. Two new cohorts of mice were trained to nose-poke for food (Figs 1D and S1A). One group was used as a basal control (T) and the second group was challenged with an additional VI-60 session (T_S). Both groups increased ANP throughout training at similar rates (ANP/min, session: *F*_5,80_ = 28, ****p < 0.0001; group: *F*_1,16_ = 0.03, *p* = 0.9; interaction, *F*_5,80_ = 0.3, *p* = 0.9; Fig 1E). The rates of magazine entries (ME) and inactive nose-pokes (INP) did not differ between groups (*p* > 0.05; S1A Fig). Akt phosphorylation was increased in the T_S group compared to T mice (pAkt/Akt: T versus T_S, ***p < 0.001; Fig 1F). In independent cohorts (S1B Fig), this activation was abolished by intra-DLS administration of the mGluR5 antagonist MPEP (3.85 ng/0.5 μl; *p* > 0.05) 30 min prior to the session, confirming that mGluR5 mediated this effect (S1C Fig).

These findings indicate that mGluR1/5 in the DLS are engaged by instrumental conditioning for food during early training stages. This aligns with evidence implicating the DLS in the acquisition and in the consolidation of stimulus-response associations that automate behavior [5,43,44].

### Contingency change reveals reduced mGluR1/5 signaling capacity in the DLS upon habit training

Next, we assessed overtraining-induced adaptations in mGluR1/5 activation. Using a variable interval reinforcement schedule as before, mice were either short-trained or overtrained to nose-poke for food [20,24] (Figs 1G and S1D). Both groups showed similar variations in performance during training (ANP/min, session: *F*_8,264_ = 72, ****p < 0.0001; group: *F*_1,33_ = 0.7, *p* = 0.4; interaction, *F*_8,264_ = 1.8, *p* = 0.07; Fig 1H). The two groups also had similar inactive nose poke (INP) and magazine entry (ME) rates (*p* > 0.05, S1E Fig).

Flexible, goal-directed control of behavior should change in response to varying action–outcome (A–O) contingencies and outcome values (expected in short-trained mice), while inflexible, habitual behavior should persist despite changes in A–O contingency and outcome devaluation (expected in overtrained mice) [20,24,38]. In a first subset of mice, we confirmed these predictions under the current experimental conditions. We used a validated post-training omission procedure that involved a shift from a positive to a negative contingency: food was delivered when mice refrained from nose-poking and was not delivered when they nose-poked (Figs 1G, 1I and S1D and S1F). In this procedure, the ability to suppress the previously learned nose-poke behavior in the face of a new contingency that no longer requires it tests the animal’s behavioral flexibility [20,24,40,45,46] and it involves changes in DLS glutamatergic signaling [24].

Short-trained mice showed goal-directed nose-poking behavior, as they had a lower ANP rate computed over the A–O negative contingency than a control session (A–O positive contingency). Conversely, overtrained mice did not adapt their behavior when the contingency was changed, indicating inflexible behavior (A–O contingency: *F*_1,16_ = 10, **p = 0.006, group: *F*_1,16_ = 3.2, *p* = 0.09, A–O contingency × interaction: *F*_1,16_ = 6.8, *p = 0.02; Fig 1I). Time courses of ANP ratio indicated a main group effect (time: *F*_5,80 _= 5, ***p = 0.0005; group: *F*_1,6 _= 6, *p = 0.03; interaction: *F*_5,80 _= 2, *p = 0.08; Fig 1I). Control and A–O reversal sessions in the two mouse cohorts yielded similar levels of INP (*p* > 0.05; S1F Fig). During the positive contingency session, both short-trained and over-trained mice earned the same number of pellets, however during the A–O reversal, short-trained mice received more pellets than over-trained mice (A–O contingency: *F*_1,16_ = 0.09, *p* = 0.76, group: *F*_1,16_ = 15.8, **p = 0.001, A–O contingency × interaction: *F*_1,16_ = 9.1, **p = 0.008; S1F Fig). This result indicates that the task outcome is significantly impacted by performance during changes in A–O, corroborating the evidence that overtraining leads to a failure in adapting behaviors to maximize rewards. In a second subset of short- and overtrained mice, we assessed the different responses to specific sensory satiety devaluation of the outcome of nose-poke behavior during training [8,20,24,47] (Figs 1G and S1D). In short-trained, but not in overtrained mice, the devalued condition resulted in a decrease in ANP rate compared to the valued condition (condition: *F*_1,15 _= 3.9, *p* = 0.09; group: *F*_1,15 _= 0.7, *p* = 0.4; condition × group interaction: *F*_1,15 _= 15.41, **p = 0.001; Fig 1J). INP rates and pellet consumption were similar in the valued and devalued conditions for both short- and overtrained mice (*p* > 0.05; S1G Fig). These results are in line with our previous work in mice subjected to comparable training conditions [24], indicating that overtrained behavior is consistent with inflexible, habitual performance.

**Fig 2:**
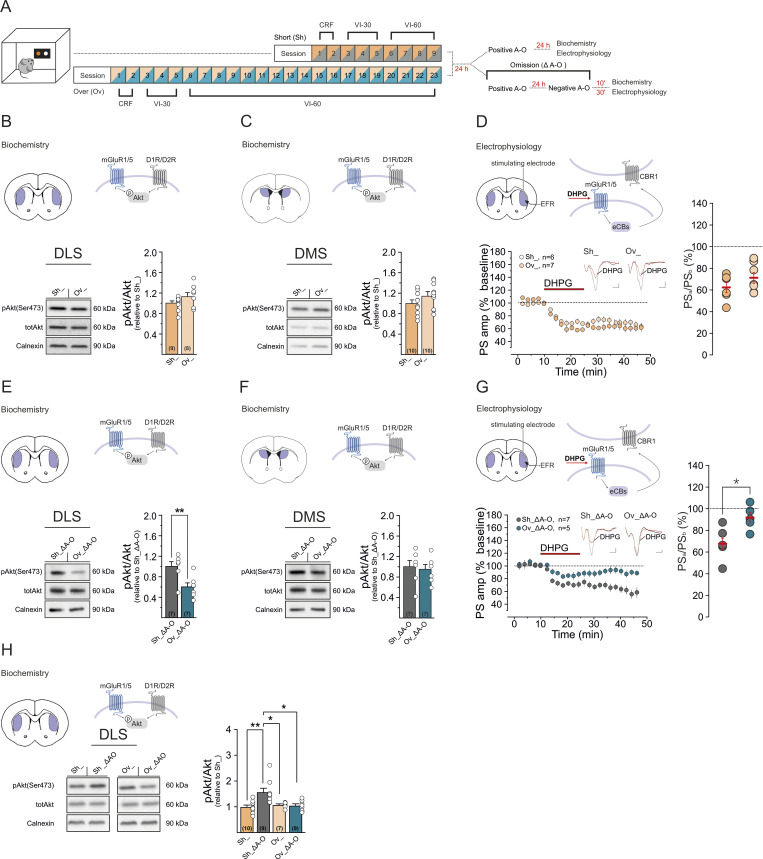
Overtrained mice show reduced signaling capacity of DLS mGluR1/5 in response to a change in the prevailing A–O contingency. **(A)** Schematic of the behavioral regimes followed by protein analysis or ex vivo electrophysiology. **(B)** Representative western blots of pAkt, totAkt, and Calnexin protein expression in the DLS of Sh_ and Ov_ mice, 24 h after the positive contingency session. Bar graphs are expression level ratios (relative to Sh_) of pAkt/totAkt (Sh__pAKT/totAKT_, 1.00 ± 0.12, *n* = 8; Ov__pAKT/totAKT_, 1.13 ± 0.08, *n* = 8; Sh__pAKT/totAKT_ *vs.* Ov_ _pAKT/totAKT_, unpaired *t* test *p* = 0.17, *t* = 1.44, *dF* = 15). *Insets*, schematic of targeted signaling proteins. **(C)** Representative western blots of pAkt, Akt, and Calnexin in the DMS of Sh_ and Ov_ mice. Bar graphs are expression level ratios (relative to Sh_) of pAkt/Akt (Sh__pAkt/Akt_: 1.00 ± 0.07, *n* = 10; Ov__pAkt/Akt_: 1.16 ± 0.08, *n* = 10; Sh__pAkt/Akt_ *vs.* Ov__pAkt/Ak_, unpaired *t* test, *p* = 0.15 *t* = 1.5, *df* = 18). **(D)** Depression of PS responses following bath application of DHPG (100 μm) in Sh_ and Ov_mice (Sh_: slices *n* = 6, mice *n* = 3; *F*_5,22_ = 28.6, ****p < 0.0001, Tukey’s *p < 0.05; Ov_: slices *n* = 7, mice *n* = 4; *F*_6,22_ = 17.5, ****p < 0.0001, Tukey’s *p < 0.05: Sh_ *vs.* Ov_, Mann–Whitney test, *p* = 0.3, *U* = 13). *Insets*, averaged recordings (5) from slices before (black line) and after DHPG application (red line). Scale bars: 0.1 mV/1 ms. **(E)** Representative western blots of pAkt, Akt, and Calnexin in the DLS of Sh_Δ A–O and Ov_Δ A–O mice, 10 min after the reward omission session. Bar graphs are expression level ratios (relative to Sh_Δ A–O) of pAkt/Akt (Sh_Δ A–O_pAkt/Akt_: 1.00 ± 0.09, *n* = 7; Ov_Δ A–O_pAkt/Akt_: 0.60 ± 0.08, *n* = 7; Sh_Δ A–O_pAkt/Akt_ *vs.* Ov_Δ A–O _pAkt/Akt_ unpaired *t* test **p = 0.006, *t* = 3.31, *df* = 12). **(F**) Representative western blots of pAkt, Akt, and Calnexin in the DMS of Sh_Δ A–O and Ov_Δ A–O mice, 10 min after omission. Bar graphs are expression level ratios (relative to Sh_Δ A–O) of pAkt/Akt (Sh_Δ A–O_pAkt/Akt_: 1.00 ± 0.12, *n* = 7; Ov_Δ A–O_pAkt/Akt_: 0.95 ± 0.09, *n* = 7; Sh_Δ A–O_pAkt/Akt_ *vs.* Ov_Δ A–O_pAkt/Ak_, unpaired *t* test, *p* = 0.74, *t* = 0.34, *df* = 12). **(G)** Depression of PS responses following bath application of DHPG (100 μm) in Sh_Δ A–O and Ov_Δ A–O mice (Sh_Δ A–O: slices *n* = 7, mice *n* = 2; *F*_6,22_ = 18, ****p < 0.0001, Tukey’s *p < 0.05; Ov_Δ A–O: slices *n* = 5, mice *n* = 2; *F*_4,22_ = 6, *p = 0.01, Tukey’s *p < 0.05: Sh_Δ A–O *vs.* Ov_Δ A–O, Mann–Whitney test, *p = 0.01, *U* = 2). *Insets*, averaged recordings (5) from slices before (black line) and after DHPG application (red line). Scale bars: 0.1 mV/1 ms. **(H)** Direct comparison of basal and Δ A–O-induced pAkt and calnexin, in short-trained and overtrained mice. Bar graphs are expression level ratios (relative to Sh_) of pAkt/Akt (Sh__pAkt/Akt_: 1.00 ± 0.07, *n* = 10; Sh_Δ A–O_pAkt/Akt_: 1.57 ± 0.15, *n* = 9; Ov__pAkt/Akt_: 1.08 ± 0.04, *n* = 7; Ov_ Δ A–O_pAkt/Akt_: 1.05 ± 0.07, *n* = 9; Kruskal–Wallis: **p = 0.0013; Sh__pAkt/Akt_ *vs*. Sh_Δ A–O_pAkt/Akt_ **p = 0.0018; Sh__pAkt/Akt_ *vs*. Ov__pAkt/Akt_ *p* > 0.99; Sh__pAkt/Akt_ *vs.* Ov_Δ A–O_pAkt/Akt_ *p* > 0.99; Sh_Δ A–O_pAkt/Akt_ *vs.* Ov__pAkt/Akt_ *p = 0.04; Sh_Δ A–O_pAkt/Akt_ vs. Ov_Δ A–O_pAkt/Akt_ *p = 0.01; Ov__pAkt/Akt_ vs. Ov_Δ A–O_pAkt/Akt_ *p* > 0.99, Dunn’s). **(B–H)** Data are mean ± SEM. Data set are available at the following link: https://doi.org/10.48557/VCAWUD.

We then assessed whether overtraining affected the basal signaling activity of mGluR1/5 by measuring pAkt protein levels in the DLS of a new cohort of short- (Sh_) and overtrained (Ov_) mice, 24 h after the positive contingency session (Figs 2A and S2A, S2B). Akt phosphorylation was not significantly different in the two groups (pAKT/totAKT: Sh_ versus Ov_, *p* > 0.05; Fig 2B). Similarly, no significant differences were detected in the adjacent DMS (pAkt/Akt: Sh_ versus Ov_ *p* > 0.05; Sh_D A–O versus Ov_D A–O *p* > 0.05; Fig 2C),

We also tested the efficiency of stimulated mGluR1/5 to engage downstream signaling in response to pharmacological activation. As a functional readout, we measured DHPG-induced long-term synaptic depression (DHPG-LTD), which in the DLS relies on mGluR1/5-mediated biosynthesis of endocannabinoids (eCBs); eCBs activate the cannabinoid receptor type 1 (CB1) expressed on corticostriatal terminals, leading to a decreased probability of glutamate release [27,48,49]. We evaluated DHPG-LTD by extracellular field recording of population spikes (PS). In both Sh_ and Ov_ mice bath application of DHPG (100 μm) induced a long-lasting (>30 min) reduction of PS amplitudes compared to baseline (Sh_, 62.12 ± 4.94%, *n* = 6, *p < 0.05; Ov_, 71.07 ± 5.10%, *n* = 7, *p < 0.05), which did not differ between the two groups (*p* > 0.05; Fig 2D). These results indicate that task overtraining affected neither basal Akt- nor pharmacological mGluR1/5-mediated LTD.

We finally assessed whether task overtraining affects mGluR1/5 activation in response to a change in A–O contingency. In different cohorts of mice that were short- and overtrained to nose-poke for food and subjected to omission learning (Sh_Δ A–O; Ov_Δ A–O; Figs 2A and S2A–S2E), we analyzed Akt pathway stimulation. pAkt levels were significantly lower in Ov_Δ A–O compared to Sh_Δ A–O mice (pAkt/Akt: Sh_Δ A–O versus Ov_Δ A–O, **p < 0.01; Fig 2E). The DLS plays a critical role in the habitual control of behavior [16], and consistent with this, changes in pAkt phosphorylation following ΔA–O were restricted to the DLS. No significant differences in pAkt levels were detected in the DMS (pAkt/Akt: Sh_Δ A–O versus Ov_Δ A–O *p* > 0.05; Fig 2F), indicating that the response was anatomically specific. DHPG-LTD in the DLS showed similar effects: the depression of PS activity in response to direct pharmacological activation of mGluR1/5 was lost in Ov_Δ A–O compared to Sh_Δ A–O mice (Sh_Δ A–O, 67.23 ± 4.95%, *n* = 7, *p < 0.05; Ov_Δ A–O, 91.37 ± 5.01%, *n* = 5, *p* > 0.05; Sh_Δ A–O versus Ov_Δ A–O, *p < 0.05; Fig 2G). No significant differences in DHPG-LTD were observed across the different mouse groups or behavioral conditions when measured in the DMS (Sh_, 73.32 ± 6.19%, *n* = 7, *p < 0.05; Ov_, 68.38 ± 4.04%, *n* = 7, *p < 0.05; Sh_ versus Ov_, *p* > 0.05; S2F Fig; Sh_Δ A–O, 66.65 ± 8.63%, *n* = 6, *p < 0.05; Ov_Δ A–O, 64.87 ± 5.87%, *n* = 8, *p < 0.05; Sh_Δ A–O versus Ov_ Δ A–O, *p* > 0.05; S2G Fig).

Finally, supporting a reduction in mGluR5 signaling capacity in response to contingency change following overtraining, comparison of pAkt levels under basal conditions and after ΔA–O showed increased phosphorylation in short-trained mice (pAkt/Akt: Sh_ versus Sh_Δ A–O, **p < 0.01), an effect absent in overtrained animals (Ov_ versus Ov_Δ A–O, *p* > 0.05; Fig 2H).

Together, these data indicate that overtraining does not affect basal mGluR1/5 activation but rather impairs this its ability to signal in response to changes in behavioral contingencies.

### Overtraining differentially modulates t-LTD in DLS indirect and direct pathway SPNs following contingency shifts

**Fig 3:**
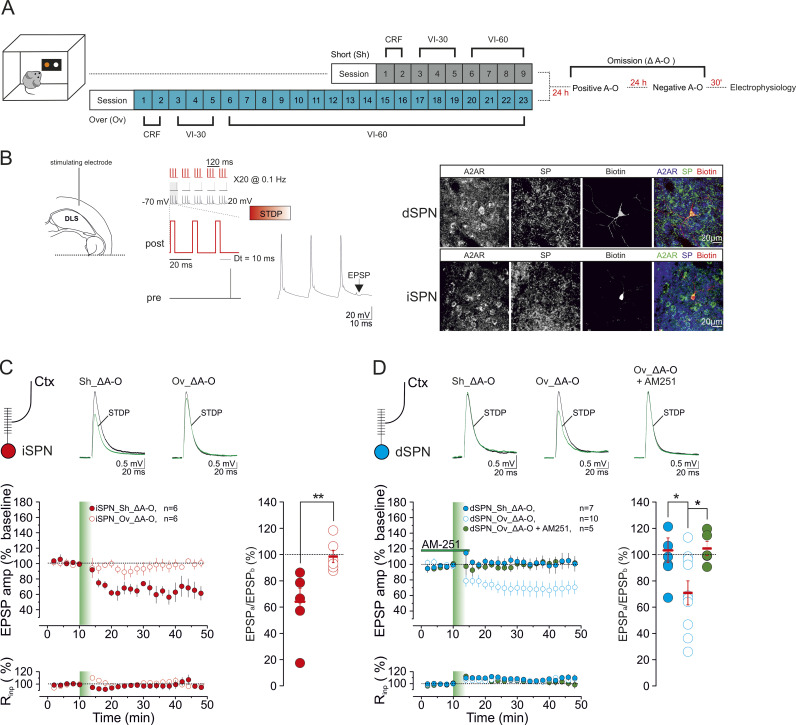
Overtrained mice show dichotomous modulation of timing-dependent synaptic depression (tLTD) in response to a change in the prevailing A–O contingency. **(A)** Schematic of the behavioral regimes followed by ex vivo electrophysiology. **(B)** (Left) Experimental configuration in horizontal brain slices containing the DLS. (Middle) The post-pre pairing protocol (negative STDP) for the induction of t-LTD. (Right) Confocal laser scanning microscopy images of triple immunofluorescence for adenosine A2A receptor (A2AR), substance P (SP), and biotin in patch-recorded neurons (scale bar: 20 µm). **(C)** In DLS iSPNs from Sh_Δ A–O mice, STDP induced a form of t-LTD (cells *n* = 6, mice *n* = 6; *F*_5,22_ = 7, ****p < 0.0001, Tukey’s, *p < 0.05) that was impaired in Ov_Δ A–O mice (cells *n* = 6, mice *n* = 6; *F*_5,22 _= 0.6, *p* = 0.7, iSPN_Sh_Δ A–O *vs.* iSPN_Ov_Δ A–O, Mann–Whitney test, **p = 0.002, U = 0). **(D)** (Left) In dSPNs, t-LTD could not be detected in Sh_Δ A–O mice (*n* = 7, mice *n* = 5; *F*_6,22_ = 0.8, *p* = 0.5), whereas it was elicited in Ov_Δ A–O mice (cells *n* = 10, mice *n* = 8; *F*_9,22_ = 6, ****p < 0.0001, Tukey’s *p < 0.05). This form of plasticity is sensitive to the bath application of the CB1R antagonist AM251 (4 μM) (cells *n* = 5, mice *n* = 4; *F*_4,22_ = 0.6, *p* = 0.6; groups comparison, *F*_2,21 _= 4.7, *p = 0.02; dSPN_Sh_Δ A–O *vs.* dSPN_Ov_Δ A–O, *p = 0.03, dSPN_Ov_Δ A–O vs. dSPN_Ov_Δ A–O + AM251, Dunnett’s *p = 0.04). In this figure, and in the analogous plots that follow throughout the manuscript, data are presented as a time course (mean ± SEM) of normalized EPSP amplitudes and normalized Rinp. The scatterplot summarizes the ratios of synaptic responses after (a) and before (b) the STDP, at the time points indicated. Insets represent superimposed averaged recordings (10 traces) before and after the delivery of the STDP protocol (vertical bar). Data set are available at the following link: https://doi.org/10.48557/VCAWUD.

At DLS corticostriatal synapses, mGluR1/5 signaling, through the activation of the eCB pathway, is a critical determinant of long-term synaptic depression that depends on the relative timing of presynaptic cortical and SPN neuronal activity (t-LTD) [50,51]. This form of plasticity can be induced experimentally on ex-vivo brain slices by preceding cortical stimulation (negative timing) with postsynaptic back-propagating action potentials in the presence of GABAergic antagonists [20,49,51,52]. While t-LTD can be reliably induced at cortico-iSPN synapses, it cannot be gated at cortico-striatal synapses on dSPNs in naive mice unless dopamine D1 receptors (D1R) are inhibited [20,51,53]. In a mouse model of cannabinoid tolerance (i.e., mice chronically exposed to the CB1R agonist Δ9-THC), the transition from goal-directed to habitual actions is marked by the loss of striatal t-LTD in iSPNs, following the downregulation and desensitization of CB1R [20]. After task overtraining, mGluR1/5 signaling is reduced in response to A–O contingency changes (Fig 2E). We, therefore, asked whether corticostriatal t-LTD was differentially affected by the omission paradigm in short-trained and overtrained mice (Sh_Δ A–O; Ov_Δ A–O; Fig 3A) and whether this effect was cell type-specific. Thirty minutes after the reward omission procedure, we recorded evoked excitatory postsynaptic potentials (EPSPs) in brain slices containing the DLS, upon electrical stimulation of deep cortical layer 5 (Fig 3B). iSPNs and dSPNs were identified by their negative resting membrane potentials, firing activity [48], and the expression of the markers adenosine receptor A2A (for iSPNs) and substance P (for dSPNs) (Fig 3B), as previously described [20,48,54].

In iSPNs from Sh_Δ A–O mice, the negative STDP paradigm induced a form of t-LTD of EPSPs (64 ± 10% of baseline, *n* = 6, *p < 0.05, Fig 3C) that is dependent on eCB signaling, as it was blocked by CB1R antagonist AM251 (4 μM) (97.0 ± 5%, *n* = 6, *p* > 0.05; iSPN_ Sh_Δ A–O versus iSPN_ Sh_Δ A–O + AM251, **p < 0.001; S3B Fig). In dSPNs from the same mouse cohort, the STDP protocol failed to trigger plasticity (103 ± 9.5%, *n* = 7, *p* > 0.05; Fig 3D). These results are consistent with many previous studies investigating t-LTD in iSPNs and dSPNs [20,51,53,54].

Ov_Δ A–O mice showed the opposite effect: t-LTD at cortico-iSPNs synapses was impaired (98.6 ± 5.0%, *n* = 6, *p* > 0.05; iSPN_Sh_Δ A–O versus iSPN_Ov_Neg; **p < 0.01, Fig 3C). t-LTD was not occluded by a prior release of eCBs during the omission procedure, as there was no significant reduction in the frequency or amplitude of miniature EPSCs (mEPCs) in mice subjected to Δ A–O (30 min after; iSPN_Δ A–O, *n* = 5), compared to a subset of overtrained mice subjected only to the positive contingency session and recorded 24 h later [(iSPN_Ov_, *n* = 6; iSPN_Ov_Δ A–O versus iSPN_Ov_; frequency (Hz) *p* > 0.05, amplitude (pA) *p* > 0.05; S3C Fig)], which suggests that basal glutamate release probability did not differ between the two mouse groups [55]. This was further supported by the analysis of synaptic variability, as there were no significant changes in the coefficient of variation (CV) of individual evoked EPSP between the two groups, as reflected by the values of calculated CV^−2^, which is a sensitive measure of presynaptic release probability (iSPN_Δ A–O, *n* = 6; iSPN_Ov_, *n* = 8; iSPN_Ov_Δ A–O versus iSPN_Ov_, *p* > 0.05; S3D Fig) [56].

In contrast, we detected a form of t-LTD at cortico-dSPN synapses in Ov_Δ A–O mice (71.0 ± 9.0%, *n* = 10, *p < 0.05, Fig 3D). This form of plasticity was CB1R-mediated, as it was sensitive to AM251 (105.0 ± 5.0%, *n* = 5, *p* > 0.05; dSPN_Sh_Δ A–O versus dSPN_Ov_Δ A–O, *p < 0.05; dSPN_Ov_Δ A–O versus dSPN_Ov_Δ A–O + AM251, *p < 0.05; Fig 3D).

In summary, while overtrained, inflexible mice show impaired eCB-mediated t-LTD in iSPNs upon omission, the same behavioral manipulation permits a form of eCB-dependent t-LTD in dSPNs, which is absent in short-trained, goal-directed animals.

### Preventing activation of mGluR5 at late training stages preserves behavioral sensitivity to changes in A–O association after overtraining, and averts cell-type specific synaptic alterations

Data so far indicate an association between overtraining-induced inflexible behavioral performance and a reduced ability of mGluR1/5 to engage downstream signaling in response to contingency changes. To determine whether these two phenomena are directly linked, we designed a homotypic rescue strategy. During prolonged training, repetitive glutamatergic stimulation of mGluR1/5 can result in downregulation of either its availability at the membrane or its coupling with the downstream signaling targets through feedback mechanisms [57–60], potentially reducing the adaptability to contingencies changes. We have previously shown that manipulation of striatal glutamatergic transmission during the late phase of training (i.e., from sessions 16–22) affects instrumental control (goal-directed versus habitual) [20,24]. We, therefore, reasoned that by hindering DLS mGluR1/5 activation during this time window, thereby preventing reduction of its signaling capacity through a homotypic process, behavioral sensitivity to changes in A–O contingencies should be preserved, and biochemical and synaptic changes in overtrained mice averted.

To test this hypothesis, we targeted the mGluR5 receptor subtype, as it plays a major role in the regulation of striatal function and SPN synaptic plasticity [35,48,50,61]. We trained mice to nose-poke for food for 15 sessions, after which we bilaterally implanted infusion cannulae in the DLS to allow local administration of MPEP (3.85 ng/0.5 μl) or vehicle. As a negative allosteric modulator of mGluR5, MPEP is expected to attenuate mGluR5 activation during late training to a level that prevents overstimulation and subsequent adaptations.

**Fig 4:**
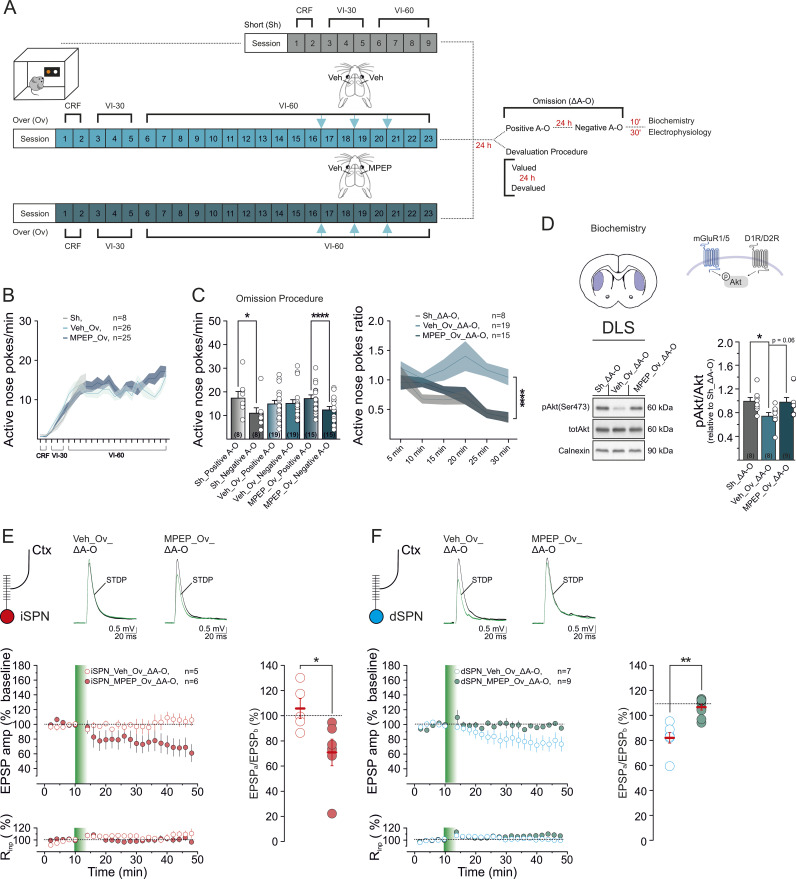
In vivo DLS infusion of the mGluR5 inhibitor MPEP at late training stages preserves behavioral sensitivity to contingency change in overtrained mice. **(A)** Schematic depicts the behavioral regimes and in vivo DLS infusions. **(B)** ANP rates during instrumental conditioning in overtrained mice injected with MPEP or its vehicle, and in the short-trained control group (ANP/min, Sh, *n* = 8; Veh_Ov, *n* = 26; MPEP_Ov, *n* = 25). **(C)** Post-training omission procedure. (Left) Comparison of ANP rates between positive and negative A–O contingency in the different experimental groups (Sh *n* = 8, positive A–O: 17.34 ± 2.84, negative A–O: 10.98 ± 2.28, Sidak ****p < 0.0001; Veh_Ov *n* = 19, positive A–O: 14.89 ± 1.5, negative A–O 15.12 ± 1.6, Sidak *p* = 0.99; MPEP_Ov *n* = 15, positive A–O: 17.13 ± 1.58, negative A–O 12.36 ± 1.47, Sidak ****p < 0.0001. (Right) Time courses of ANP ratios (ANP rates under negative A–O/ANP rates under positive A–O) in the three mouse cohorts. **(D)** Representative western blots of pAkt, Akt and Calnexin in a subset of short-trained- (*n* = 8) and overtrained mice treated with either vehicle (*n* = 8) or MPEP (*n* = 9), 10 min after the omission procedure. Bar graphs are expression level ratios pAkt/Akt (relative to Sh_Δ A–O; Sh_Δ A–O _pAkt/Akt_,1 ± 0.07; Veh_Ov_Δ A–O _pAkt/Akt_, 0.7 ± 0.06; MPEP_Ov_Δ A–O_pAkt/Akt_, 0.98 ± 0.08, *n* = 9; *F*_2,22_ = 4, *p = 0.03; Sh_Δ A–O_pAkt/Akt_ vs. Veh_Ov_Δ A–O_pAkt/Akt_, Tukey’s *p = 0.04; Sh_Δ A–O_pAkt/Akt_ vs. MPEP_Ov_Δ A–O_pAkt/Akt_, Tukey’s *p* = 0.96; Veh_Ov_Δ A–O_pAkt/Akt_ vs. MPEP_Ov_Δ A–O_pAkt/Akt_, *p* = 0.06). **(E–F)** t-LTD in DLS iSPNs **(E)** or DLS dSPNs **(F)** from overtrained mice administered with MPEP or vehicle, and subjected to Δ A–O (iSPN_Veh_Ov_Δ A–O: cells *n* = 5, mice *n* = 5, *F*_4,22 _= 0.9, *p* = 0.5; iSPN_MPEP_Ov_Δ A–O: cells *n* = 6, mice *n* = 6, *F*_5,22_ = 4.4, ****p < 0.0001, Tukey’s *p < 0.05; iSPN_Veh_Ov_Δ A–O *vs.* iSPN_MPEP_Ov_Δ A–O, Mann–Whitney test, *p = 0.02, *U* = 2; dSPN_Veh_Ov_Δ A–O: cells *n* = 7, mice *n* = 7, *F*_6,22 _= 3.6, ****p < 0.0001, Tukey’s *p < 0.05; dSPN_MPEP_Ov_Δ A–O: cells *n* = 9, mice *n* = 9; *F*_8,22 _= 1, *p* = 0.4; dSPN_Veh_Ov_Δ A–O vs. dSPN_MPEP_Ov_Δ A–O, *t* test, **p = 0.003, *t* = 3.652, *dF* = 14). Data set are available at the following link: https://doi.org/10.48557/VCAWUD.

After the mice recovered from surgery, their training resumed for two sessions. We then administered MPEP or vehicle for three consecutive days. In parallel, a naive mouse group (Sh) was subjected to a short training to control for behavioral sensitivity to contingency change (Figs 4A and S4A). In the overtrained mice, administering MPEP had no effect on active nose-poke rates across training sessions, compared to vehicle (Sh, *n* = 8, Veh_Ov, *n* = 26, MPEP_Ov, *n* = 25; ANP/min: session: *F*_8,448_ = 92, ****p < 0.0001_;_ group: *F*_2,56_ = 0.5, *p* = 0.6; interaction: *F*_8,448_ = 1.5, *p* = 0.09; Fig 4B) nor on ME or INP rates (*p* > 0.05, S4B Fig). In contrast, when tested in a subset of mice from the three experimental groups during the post-training omission procedure, the administration of MPEP specifically affected behavioral response to changes in the A–O contingency (Sh_Δ A–O *n* = 8, Veh_Ov_Δ A–O, *n* = 19, MPEP_Ov_Δ A–O *n* = 15; ANP/min, session: *F*_1,39_ = 43.29, ****p < 0.0001, group: *F*_2,39_ = 0.05, *p* = 0.95, post-training session × interaction, *F*_2,39_ = 14.84, ****p < 0.0001; Fig 4C). The time course analysis of ANP ratios reveals a main group effect between the three mouse cohorts (time: *F*_5,195_ = 5, ***p = 0.0002, group: *F*_2,39_ = 12.4, ****p < 0.0001, interaction: *F*_10,195_ = 1.7, *p = 0.08; Fig 4C). There were no differences in INP rates between control and A–O reversal sessions among mouse groups (*p* > 0.05; S4C Fig). Consistent with more flexible behavioral control, overtrained mice treated with MPEP received more pellets than the vehicle group during A–O reversal. The number of reinforcers obtained during the control session did not differ among groups (A–O contingency: *F*_1,39_ = 47.42, ****p < 0.0001; group: *F*_2,39_ = 9.29, ***p = 0.0005; A–O contingency × group interaction, *F*_2,39_ = 7.15, **p = 0.002; S4C Fig).

Regulation of mGluR5-mediated signaling might be less important for animals to adapt to outcome devaluation, as this behavioral domain does not require adapting to novel environmental contingencies. Consistent with this, the loss of sensitivity to devaluation displayed by a subset of overtrained animals is not rescued by MPEP administration (Veh_Ov *n* = 7; MPEP_Ov *n* = 10; condition: *F*_1,15_ = 0.02, *p* = 0.89; group: *F*_1,15_ = 1.15, *p* = 0.3; condition × group interaction, *F*_1,15_ = 0.53, *p* = 0.47; S4D Fig). INP rates and pellet consumption were comparable in the valued and devalued conditions in the two mouse groups (*p* > 0.05; S4D Fig).

Preventing activation of mGluR5 at late training stages rescued pAkt to levels comparable to those in short-trained mice upon omission. Specifically, when compared to pAkt protein levels in Sh_Δ A–O mice (*n* = 8), values were significantly lower in Ov_Δ A–O mice injected with vehicle (Veh_Ov_Δ A–O *n* = 8), but did not differ in the group infused with MPEP (MPEP_Ov_Δ A–O *n* = 9; pAkt/Akt: *F*_2,22_ = 4, *p* = 0.03; Sh_Δ A–O_pAkt/Akt_ *versus* Veh_Ov_Δ A–O_pAkt/Akt_, *p < 0.05; Sh_Δ A–O_pAkt/Akt_ *versus* MPEP_Δ A–O_pAkt/Akt_, *p* > 0.05; Fig 4D).

In vivo mGluR5 antagonism during late training stages also restored t-LTD at cortico-iSPN synapses (iSPN_Ov_Veh_Δ A–O, 106.0 ± 8.0%, *n* = 5, *p* > 0.05; iSPN_MPEP_Ov_Δ A–O, 71.0 ± 11.0%, *n* = 6, *p < 0.05; iSPN_Ov_Veh_Δ A–O versus iSPN_MPEP_Ov_Δ A–O, *p < 0.05; Fig 4E), and prevented plasticity at cortico-dSPN synapses in the DLS of MPEP_Ov_Δ A–O mice (dSPN_MPEP_Ov_Δ A–O, 98.0 ± 2.4%, *n* = 9, *p* > 0.05) compared to Veh_Ov_Δ A–O animals (dSPN_Veh_Ov_Δ A–O, 81.0 ± 4.9%, *n* = 7, *p < 0.05; dSPN_MPEP_Ov_Δ A–O versus dSPN_Veh_Ov_Δ A–O, **p < 0.01; Fig 4F).

Together, these results show that preventing the repeated stimulation of mGluR5 in the DLS during the late stage of task overtraining preserves the ability to update changes in A–O contingencies and prevents cell type-specific changes in mGluR5-eCB-mediated t-LTD through a homotypic process.

### mGluR5 activation biases distinct intracellular mechanisms to regulate t-LTD in iSPNs and dSPNs

The cell type-specific effects on t-LTD upon omission learning in iSPNs and dSPNs suggest that mGluR1/5 may bias different signaling mechanisms in the two neuronal subpopulations. During negative STDP in iSPNs, the synergistic activation of mGluR1/5 and dopamine D2 receptors, as well as Ca^2+^ entry via voltage-gated calcium channels (VGCCs), converges on Gq-PLCβ signaling to trigger the biosynthesis of eCBs, ultimately leading to depressed corticostriatal inputs [20,49,51]. In line with these results, the altered signaling capability of mGluR1/5 signaling in Ov_Δ A–O mice is associated with the loss of eCB-mediated t-LTD in iSPNs (Figs 2G, 2H, and 3C).

In dSPNs, the same behavioral manipulation is associated with a form of eCB-LTD (Fig 3D). This raises the possibility that in this cell type, mGluR1/5 biases intracellular pathways that counteract the induction of t-LTD. In cultured striatal neurons, mGluR5 can constitutively bind to calcium/calmodulin-dependent protein kinase II (CaMKII) in its inactive form. Activation of mGluR5 stimulates Ca^2+^ signaling, leading CaMKII to dissociate from the receptor and bind to the NMDAR GluN2B subunit. This enables CaMKII to phosphorylate GluN2B, ultimately increasing GluN2B availability at the membrane [33]. In brain regions other than the striatum, the CaMKII-GluN2B complex favors the potentiation of synaptic strength [62]. Activated striatal CaMKII also inhibits the enzyme diacylglycerol lipase (DGL), which is key for eCB biosynthesis [63–65].

**Fig 5:**
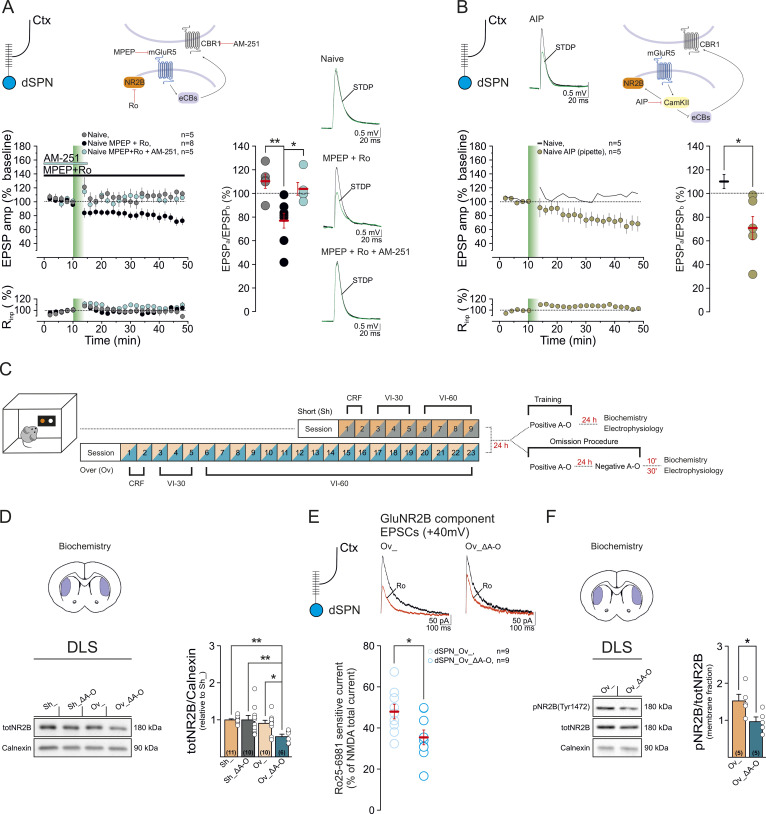
mGluR5 biases different intracellular pathways in iSPNs and dSPNs to regulate DLS t-LTD. **(A–B)** Plasticity of EPSPs in DLS dSPNs from naive mice in response to negative STDP (dSPN_Naïve, cells *n* = 5, mice *n* = 4, *F*_4,22 _= 0.9, *p* = 0.5). **(A)** Co-application of the mGluR5 allosteric inhibitor MPEP (10 μM) with the GluN2B antagonist Ro-256981 (1 μM) gated a form of t-LTD (dSPN_Naïve + MPEP + Ro, cells *n* = 8, mice *n* = 6, *F*_7,22_ = 7, ****p < 0.0001, Tukey’s *p < 0.05) that was blocked by AM251 (4 μM; dSPN_Naïve + MPEP + Ro + AM251, cells *n* = 5, mice *n* = 3, *F*_4,22 _= 0.9, *p* = 0.4; groups comparison, *F*_2,17_ = 9, **p = 0.003; dSPN_Naïve versus dSPN_Naïve + MPEP + Ro, Tukey’s **p < 0.01, dSPN_Naïve versus dSPN_Naïve + MPEP + Ro + AM251, Tukey’s *p* > 0.05, dSPN_Naïve + MPEP + Ro versus dSPN_Naïve + MPEP + Ro + AM251, Tukey’s *p < 0.05). **(B)** The intracellular inclusion of the CaMKII inhibitor AIP (10 μM) enabled t-LTD at cortico-dSPN synapses (dSPN_Naïve + AIP, cells *n* = 6, mice *n* = 3, *F*_5,22_ = 4.9, ****p < 0.0001, Tukey’s *p < 0.05; dSPN_Naïve versus dSPN_Naïve + AIP, Mann–Whitney test, *p = 0.02, *U* = 2). The solid black line (average) is the time course from Fig 5A, and is reported here for comparison. **(C)** Schematic of behavioral training paradigms followed by protein analysis, and ex vivo electrophysiology. **(D)** Representative western blots of GluN2B and Calnexin in the DLS of short- and overtrained mice (Sh_ and Ov_), and in short- and overtrained mice after omission (Sh_Δ Α−Ο and Ov_Δ Α−Ο). Bar graphs are expression level ratios (relative to Sh_) of GluN2B/calnexin (Sh__totNR2B/Calnexin_, 1.00 ± 0.03, *n* = 11; Sh_Δ Α−Ο_totNR2B/Calnexin_, 1.00 ± 0.12, *n* = 10; Ov__totNR2B/Calnexin_, 0.91 ± 0.08, *n* = 10; Ov_Δ Α−Ο_totNR2B/Calnexin_, 0.55 ± 0.07, *n* = 6; *F*_3,33_ = 5, **p = 0.005; Sh__totNR2B/Calnexin_ versus Sh_Δ Α−Ο_totNR2B/Calnexin_, Tukey’s *p* > 0.99; Sh__totNR2B/Calnexin_ versus Ov__totNR2B/Calnexin_, Tukey’s *p* = 0.83; Sh__totNR2B/Calnexin_ versus Ov_Δ Α−Ο_totNR2B/Calnexin_, Tukey’s **p = 0.006; Ov__totNR2B/Calnexin_ versus Ov_Δ Α−Ο_totNR2B/Calnexin_, Tukey’s *p = 0.04; Ov__totNR2B/Calnexin_ versus Sh_Δ Α−Ο_totNR2B/Calnexin_, Tukey’s *p* = 0.84; Sh_Δ Α−Ο_totNR2B/Calnexin_ versus Ov_Δ Α−Ο_totNR2B/Calnexin_ Tukey **p = 0.007). **(E)** (Left) Sample traces of NMDA-mediated EPSCs recorded before (black line) and after (red line) application of Ro-256981 (1 μM). (Right) Scatterplot summarizes the magnitude of the Ro-256981-sensitive component (% of total NMDA current), referred to as the GluN2B component (dSPN_Ov_ versus dSPN_Ov_Δ Α−Ο, *t* test, *p = 0.02, *t* = 2.485 *dF* = 16). **(F)** Changes in the membrane fraction of GluN2B phosphorylation at the Y1472 site in Ov_Δ Α−Ο compared to Ov_ mice (Ov__pNR2B/totNR2B_, 1.53 ± 0.18, *n* = 5, Ov_Δ Α−Ο_pNR2B/totNR2B_, 0.97 ± 0.13, *n* = 5; Ov__pNR2B/totNR2B_ versus Ov_Δ Α−Ο_pNR2B/totNR2B_, *t* test, *p = 0.03, *t* = 2.57, *df* = 8).

We hypothesized that in dSPNs, mGluR5, or distinct pools of mGluR5 compared to iSPNs, preferentially signal through a CaMKII-GluN2B pathway in response to negative STDP, which counteracts eCB-tLTD induction. If this is true, the concomitant inhibition of mGluR5 and GluN2B should allow eCB t-LTD to occur. Indeed, compared to control conditions (dSPN_Naïve, *n* = 5; 110.0 ± 6.0%, *p* > 0.05), co-application of the mGluR5 antagonist MPEP (10 μM) and the GluN2B blocker Ro-256981 (Ro, 1 μM) during negative STDP resulted in t-LTD at cortico-dSPN synapses of naive mice (dSPN_Naïve + MPEP + Ro, *n* = 8; 76.6 ± 6.3%, *p < 0.05). This form of t-LTD was blocked by the CB1 antagonist AM251 (4 μM) (dSPN_Naïve + MPEP + Ro + AM251, *n* = 5; 104.3 ± 5.3%, *p* > 0.05; dSPN_Naïve *versus* dSPN_Naïve + MPEP + Ro, **p < 0.01; dSPN_Naïve *versus* dSPN_Naïve + MPEP +Ro + AM251, *p* > 0.05, dSPN_Naïve + MPEP + Ro versus dSPN_Naïve + MPEP + Ro + AM251, *p < 0.05; Fig 5A) and by the L-Type VGCC blocker nimodipine (10 μM) (dSPN_Naïve + MPEP + Ro + Nimodipine, *n* = 6; 102.0 ± 6.4%, *p* > 0.05; dSPN_Naïve + MPEP + Ro *versus* dSPN_Naïve + MPEP + Ro + Nimodipine, **p < 0.01; S5A Fig), confirming that plasticity relies on eCB release. Ruling out the potential effect of MPEP on NMDA function [66], application of either MPEP or Ro alone during negative STDP failed to induce t-LTD (dSPN_Naïve + MPEP, *n* = 5; 98.3 ± 7.0%, *p* > 0.05; dSPN_Naïve + Ro, *n* = 5; 98.7 ± 3.7%, *p* > 0.05; S5B Fig). In contrast, including the CaMKII inhibitor Autocamtide-2-Related Inhibitory Peptide (AIP; 10 μM) in the postsynaptic dSPN neuron gated plasticity (dSPN_Naïve + AIP, *n* = 6; 70.2 ± 10.0%, *p < 0.05; dSPN_Naïve *versus* dSPN_Naïve + AIP, *p < 0.05; Fig 5B). This indicates that the activation of CaMKII during negative STDP is a key counteracting mechanism of t-LTD in dSPNs. Furthermore, the activation of L-Type VGCCs is sufficient to enable eCB-LTD in dSPNs when mGluR5 and GluN2B are simultaneously blocked.

These findings suggest that, in overtrained mice, adaptations in mGluR1/5 signaling capacity upon omission may result in less active CaMKII during the negative STDP, thereby reducing the availability of GluN2B at the membrane and ultimately promoting eCB-mediated t-LTD in dSPNs. To test this hypothesis, we first compared basal levels of GluN2B protein in the DLS of short- and overtrained mice (Sh_ and Ov_, Fig 5C and 5D) with GluN2B protein levels in short- and overtrained mice after omission (Sh_Δ A–O and Ov_Δ A–O; protein samples from training groups described in Figs S2B and 5C and 5D). As predicted, GluN2B protein expression was reduced in Ov_Δ A–O compared to the other cohorts (Sh_ *n* = 11, Ov_ *n* = 10, Sh_Δ A–O, *n* = 10, Ov_Δ A–O = 6; **p < 0.01; Ov_Δ A–O *versus* Sh_, **p < 0.01; Ov_Δ A–O *versus* Sh_Δ A–O, **p < 0.01; Ov_Δ A–O *versus* Ov_, *p < 0.05; Fig 5D). We then confirmed reduced functional expression of GluN2B at cortico-dSPN synapses by measuring the Ro-sensitive component of NMDA currents in a subset of Ov_ and Ov_Δ A–O mice. DLS dSPNs were held at a potential of +40 mV to relieve the Mg^2+^ blockade and NMDA EPSCs were isolated in the presence of NBQX (20 μM) and GBZ (10 μM). Perfusion of Ro-256981 (1 μM) caused a substantial decrease in NMDA EPSCs (Fig 5E, referred to as GluN2B component). In Ov_, the GluN2B component was 48 ± 4% of the total NMDA current (*n* = 9). Consistent with changes in protein expression upon omission, we observed a significant decrease in the GluN2B-mediated component in Ov_Δ A–O mice (*n* = 9; Ov_Δ A–O, 35 ± 4% of total currents; Ov_ *versus* Ov_Δ A–O, *p < 0.05; Fig 5E). Phosphorylation of GluN2B at site Y1472 disrupts the binding to the AP-2 clathrin-associated adaptor protein complex, which targets proteins for endocytosis [67]. This raises the possibility that in response to a change in contingency following reward omission, reduced GluN2B Y1472 phosphorylation promotes GluN2B endocytosis, thereby driving the decrease in GluN2B-containing NMDAR-mediated transmission in Ov_Δ A–O mice (Fig 5E). Consistent with this model, we found that in membrane fraction, GluN2B phosphorylation at Y1472 is significantly less in the Ov_Δ A–O than the Ov_ cohort (Ov_T, 1.53 ± 0.18, *n* = 5; Ov_Neg, 0.97 ± 0.13, *n* = 5; *p < 0.05; Fig 5F).

In summary, these observations indicate that mGluR5 signals to different intracellular mechanisms to regulate t-LTD in iSPNs and dSPNs.

## Discussion

This study establishes a key molecular mechanism that supports the loss of behavioral flexibility after overtraining of a contingent operant task: reduced adaptive signaling capacity of DLS mGluR5 in response to changes in the prevailing A–O contingency. We recognize that these findings may be specific to the male P45–P60 mice used in this study and caution should be exercised when extrapolating these results to females or different developmental stages; further investigations are warranted.

### Molecular signature of behavioral inflexibility

To assess the functionality of DLS mGluR1/5, we examined the downstream Akt pathway and the eCB system (i.e., eCB-mediated LTD) [39,68]. We found that task overtraining did not affect basal Akt levels or pharmacological mGluR1/5-mediated LTD. However, in overtrained mice challenged with a change in the prevailing A–O association, we observed lower pAkt protein levels compared to short-trained mice under the same condition (Fig 2). Direct comparison of pAkt levels in short- and overtrained mice under basal conditions and following ΔA–O (Fig 2) points to a lack of activation of Akt in response to contingency change as a consequence of overtraining rather than a general reduction in phosphorylation. This could indicate a reduced ability of mGluR5 to engage downstream signaling, although potential contributions from concurrent adaptations in dopaminergic signaling cannot be excluded [69]. Likewise, the ability to release eCBs and to depress cortical inputs in response to the direct pharmacological stimulation of mGluR1/5 was reduced in response to the same behavioral manipulation (i.e., omission) in overtrained, inflexible mice compared to short-trained, goal-directed animals (Fig 2). These results raise the possibility that overtraining may bias DLS circuits toward a diminished molecular responsiveness to new contingencies, thereby favoring behavioral inflexibility. This fits the hypothesis that mGluR1/5 can act as a substrate for adaptive molecular processes that underlie the loss of flexible, goal-directed control of behavior. We speculate that, over the course of instrumental learning, mGluR1/5 may become less efficient in transducing the sensory-motor and motivational information conveyed by cortical and dopaminergic inputs, potentially representing a molecular signature of behavioral inflexibility.

Multiple mechanisms may regulate the activity of mGluR1/5 during repeated instrumental conditioning. Numerous second messenger-dependent protein kinases mediate its activity-dependent desensitization and internalization. Activating mGluR1/5 triggers the release of Ca^2+^ from intracellular stores and the activation of protein kinase C (PKC). Through a feedback mechanism, PKC activation downregulates mGluR1/5 availability at the membrane [58–60,70,71]. The mGluR5 subtype’s trafficking and signaling properties are also regulated by the Homer protein H1a, whose expression is activity-dependent [72]. H1a disrupts macromolecular signaling complexes formed by mGluR5 and downstream partners. On the other hand, the phosphorylation of H1a binding sites on mGluR5, following dopamine D1 receptor activation, connects neuronal activity and dopaminergic inputs to NMDA current potentiation [73]. Thus, mGluR5-H1a interaction may provide a means for metaplasticity during the repeated encoding of reward-related experience [73,74]. This interaction might also apply to the shift from goal-directed performance to inflexible behavior after overtraining. Notably, in the early phase of training, instrumental learning appears to be dependent on dopamine D1 receptor activation, but after overtraining, it becomes dopamine-independent [75–77]. This shift occurs in parallel with neuroadaptations of key components of the dopaminergic system, including changes in DLS dopamine receptor expression [78,79].

We have recently shown that overtraining-induced upregulation of the astrocytic glutamate transporter EAAT2 in the DLS negatively interferes with the ability to encode changes in A–O association. Astrocytic EAAT2 contributes to the regulation of glutamate spillover between synapses [80,81], thereby to glutamate receptor activation in response to specific presynaptic activity patterns [82,83]. Early in learning, there might be more corticostriatal DLS ‘neuronal ensemble’ activity, leading to greater glutamate release and spillover, which activates more mGluR5 receptors. This activity might be refined to fewer inputs and smaller ensembles as training progresses, resulting in less activation of mGluR5 receptors by glutamate, and reduced downstream signaling activation. Thus, upregulation of EAAT2 and reduced glutamate spillover in overtrained mice may also contribute to impaired mGluR1/5 activation. In this study, we did not address the precise mechanisms responsible for the overtraining-induced mGluR1/5 adaptations, which require future investigation. Nevertheless, the evidence demonstrates that interfering with mGluR5 activation by using the negative allosteric modulator MPEP at late training stages restores both receptor downstream signaling capacity and aspects of goal-directed behavior (i.e., sensitivity to contingency change) (Fig 4). If reduced glutamate spillover was the primary reason for overtraining-induced mGluR1/5 and behavioral adaptations, MPEP treatment would have a minimal impact because of the already low levels of glutamate available to activate mGluR5 in this scenario. In the biochemical experiments described in Fig 4D, the statistical comparison between vehicle- and MPEP-treated overtrained mice yielded a *p*-value of 0.06, indicating a trend toward significance that is consistent with the expected rescue effect. While this result does not reach conventional significance, it is consistent with our proposed model. These findings should be interpreted with caution, and further experiments will be necessary to validate this interpretation.

### Dichotomous modulation of t-LTD in iSPNs and dSPNs in instrumental control response

One of the fundamental points of novelty of our study is that restoring the mGluR5-dependent signaling capacity also averts the dichotomous modulation of t-LTD in iSPNs and dSPNs that occurs in overtrained mice when challenged with a reversal of a previously learned A–O association. That is, inflexible behavioral performance in overtrained mice is associated with impaired eCB-mediated t-LTD at cortico-iSPN synapses and with the appearance of a form of eCB-t-LTD in dSPNs, which is absent in both goal-directed (short-trained) and naive animals. It has been proposed that for appropriate action control, iSPN and dSPN activity must be coordinated, with dSPNs functioning to select the desired motor program, and iSPNs inhibiting competing or extraneous responses [84–86]. During instrumental learning, negative STDP would occur in those iSPN and dSPNs that are not stimulated by a specific cortical ensemble conveying the relevant sensory-motor signal to encode A–O association [87]. Depressing a subset of iSPNs and counteracting t-LTD in dSPNs in response to negative STDP may help reduce the impact of noise on the prevailing A–O representation. This mechanism would be consistent with the notion that increased cortico-dSPN connectivity is critical for the retention of task-specific information that is used to improve performance on future tasks [88]. During the omission procedure, which represents a source of negative reward prediction error and pause in striatal dopamine release [1], mice must learn to refrain from nose-poking to receive a food reward (i.e., withhold an action that is no longer related to reward) by suppressing the previously learned A–O, ultimately executing an alternative behavioral strategy [89,90]. An intriguing possibility is that the regulation of negative STDP in iSPNs and dSPNs may differ between omission and instrumental learning, potentially reflecting distinct synaptic plasticity mechanisms engaged by these behavioral contexts. Ex vivo studies indicate that while t-LTD in iSPNs requires dopaminergic signaling via dopamine D2 receptors, t-LTD at dSPN synapses relies on the absence of signaling at dopamine D1 receptors [49,51]. Specifically, in iSPNs, dopamine D2 receptor activation favors the mobilization of eCBs by disinhibiting mGluR5-Gq-PLCβ signaling [64]. This occurs through the D2-mediated inhibition of G-protein signaling 4 (RGS4), which is normally activated by PKA [91]. In dSPNs, the inhibition of RGS4, and thereby the induction of eCB-mediated t-LTD, requires either the activation of the Gi-coupled muscarinic M4 receptor or a lack of signaling at the Gs-coupled dopamine D1 receptor [52]. In this study, we revealed additional modulation mechanisms, such as mGluR5 activation during negative STDP biasing CaMKII-GluN2B signaling and counteracting t-LTD induction. Consistent with this, the concurrent inhibition of mGluR5 and GluN2B during negative STDP permits a form of eCB-t-LTD in dSPNs. This effect can be reproduced by inhibiting CaMKII activation in the postsynaptic dSPN in naive mice, or by subjecting mice to overtraining followed by omission. The omission procedure also downregulates GluN2B functional expression in overtrained mice. We propose a model in which overtraining primes the DLS mGluR5, reducing its capacity to engage downstream signaling cascades. This reduction, together with rapid GluN2B downregulation following changes in A–O, enables t-LTD in dSPNs. The model posits that although the enzymatic machinery for eCB synthesis (via PLCβ and DGL) is shared across SPNs, its functional accessibility is dynamically gated in dSPNs by mGluR5–CaMKII signaling. A different level of mGluR 1/5 engagement, secondary to overtraining-induced astrocytic EAAT2 activity [24] and tripartite-synapse-specific changes in EAAT2 signaling may also contribute to dichotomous modulation of t-LTD in iSPNs and dSPNs. During the omission procedure, this molecular scenario might affect the coordinated output of iSPNs and dSPNs in the DLS or the relative timing of the two pathways’ activity in response to cortical stimulation [38]. In overtrained mice upon omission, we found that restoring t-LTD in iSPNs and impairing this form of plasticity in dSPNs by manipulating mGluR5 signaling is associated with the retention of behavioral sensitivity to contingency reversal. Notably, downregulation of GluN2B, and thereby t-LTD at DLS cortico-dSPN synapses, occurred selectively in overtrained but not in short-trained animals. These results are in line with the proposed opposing roles of DLS dSPNs and iSPNs in goal-directed and habitual responding with inhibition of the former that prevents the learning of new contingencies (i.e., flexible behavior) and the latter that suppresses automatic responding (i.e., inflexible behavior) [92].

### Implications of molecular plasticity of mGluR5 signaling for psychiatric diseases

Our findings also provide mechanistic support for targeting mGluR5, or downstream signaling partners, in psychiatric conditions characterized by over-reliance on habitual circuitry and behavioral inflexibility. These include obsessive-compulsive disorder (OCD), Fragile-X syndrome, and autism spectrum disorders (ASD). In mouse models of these pathologies, evidence points to increased striatal mGluR5-mediated signaling and an altered mGluR5-Homer scaffold [61,93,94]. Consistent with this evidence, mGluR5 antagonists can reduce OCD- and ASD-associated repetitive behaviors, which can be considered expressions of excessive and maladaptive behavioral inflexibility [95]. Nevertheless, these psychiatric conditions are characterized by complex motor and cognitive symptomatology, and animal models show several circuit phenotypes. This complexity makes it difficult to establish causalities between a common molecular dysfunction that leads to defined synaptic alterations, and co-morbidities in specific behavioral domains. The direct relationship between the molecular plasticity of mGluR5 in DLS iSPNs and dSPNs and the animal’s inability to revert to previously learned A–O association in normal habits helps us to better understand disorders characterized by an altered balance between cognitive/behavioral flexibility and fixity.

## Materials and methods

### Experimental procedures and experimental design

All procedures involving animals were carried out in accordance with the Italian Ministry of Health’s directives (D.lgs. 116/1992 and D.lgs 26/2014) regulating animal research (n° 242/2013-B; n° 455/2016-PR; n° 905/2021-PR; n° 548/2024-PR).

### Drugs

AM251, (RS)-3,5-DHPG, Methyl-6-(phenylethynyl)pyridine hydrochloride (MPEP), NBQX disodium salt, Ro25-6981 maleate, SR 95531 hydrobromide (Gabazine), tetrodotoxin (TTx) and Nimodipine were purchased from Tocris Bioscience (Avonmouth, UK). Nimodipine and Autocamptide-2 Related Inhibitor Peptide (AIP) were purchased from Sigma-Aldrich S.r.l. (Milan, Italy). For the electrophysiological experiments, stock solutions of AM251, Methyl-6-(phenylethynyl)pyridine hydrochloride (MPEP), and Nimodipine were prepared in DMSO. Dilutions to final concentrations were made just before the start of each experiment in oxygenated aCSF. Control solutions for these experiments always contained the corresponding DMSO concentration (up to 0.1%).

### Animals

C57BL6J mice (male, postnatal day – PND45-60) were housed in a controlled environment, on a 12 h light/dark cycle, with free access to water and/or food depending on each condition.

### Behavioral experiments

#### Instrumental learning.

Behavioral training and testing were performed in operant chambers (17.8 cm × 15.2 cm × 18.4 cm) housed within sound-attenuating chambers and equipped with two holes on either side of the food magazine (Med-associates, St Albans, VT). Mice were trained to nose-poke in one of two holes to obtain two reinforcers, either chocolate (F0 5301, Bilaney, UK) or sucrose pellets (F0 5684, Bilaney, UK). One reinforcer was delivered in the operant chamber contingent upon nose poking, into the magazine through a pellet dispenser. Magazine entries were recorded using an infrared beam. The reinforcer and nose poke hole were counterbalanced across groups. Before training, mice were food deprived, as to maintain 90% of their feeding body weight. Mice were fed daily after the training session. Initial nose-poke training consisted of two consecutive daily sessions of continuous reinforcement (CRF), during which mice received a reinforcer for each nose poke. The session ended after 10 rewards. After the CRF sessions, mice were trained on variable interval (VI) schedules [8,40], in which active nose pokes were reinforced after variable time intervals that lasted on average 30 s (VI-30) or 60 s (VI-60), and ended after 20 reinforcers. After three daily VI-30 sessions, mice were either short-trained for four daily VI-60 sessions or overtrained for 18 VI-60 sessions [24].

#### Post-training omission procedure.

The omission test started one day after instrumental training and lasted two days. On day 1, mice were exposed to a 30 min control session under the prevailing, positive action–outcome contingency (nose poking leads to reinforcer delivery). On day 2, mice were subjected to a 30 min session in which the previously learned A–O contingency was changed (negative contingency). That is, the pellet was delivered every 20 s without nose poke, but each nose poke would reset the counter and delay the food delivery. Thus, the new contingency does not require mice to learn a new behavior; instead, they must learn to withhold a behavior to maximize reward**.** The rates of active nose poke (ANP) under the two different A–O contingencies (negative A–O/positive A–O) were used to determine behavioral flexibility [20,24,40,45].

#### Post-training devaluation procedure.

In the devaluation procedure, the outcome of nose poking (reinforcer delivery) was devalued using sensory-specific satiety [8,20,24]. The devaluation test started 24 h after the last training session and lasted 2 days. On each day, mice were exposed *ad libitum* to one of the reinforcers (chocolate or sucrose) for 60 min in a separate cage. On day 1, mice were given the reinforcer, previously earned by nose poking (devalued condition); on day 2 mice received the reinforcers, previously available in their home cages during training (valued condition). The order of the valued and devalued conditions was randomized. Immediately after each feeding session, the mouse underwent a 5 min extinction test in the operant chamber, during which no reinforcer was delivered. The numbers of nose pokes into the active hole under the valued and devalued conditions were compared.

### In vivo MPEP brain infusion

After the 4 VI-60 session, a subset of mice was implanted with iron cannulae (Ø 0.50/0.25 (external/internal) × 7 ± 0.05 mm, 26G, Unimed Switzerland). Cannulae were lowered in the dorsolateral striatum (DLS) at AP + 0.5, ML ± 2.7, DV −2.4 from the bregma. Three self-tapping iron screws approximately 2 mm long (FST, Germany) were previously screwed into the skull to avoid touching the surface of the brain, and dental cement (AgnThos) was used to cover the skull including the screws, maintaining the cannulae in place. The cannulae were then covered with a metal tube (Ø 0.25/0.12 (external/internal) × 25 ± 0.20 mm, 28G, Unimed Switzerland) cut and bent to fill the 7 mm cannula and to be partially included in the dental cement. This protected the brain and prevented clogging of the cannulae. Behavioral training was started again 4 days after surgery. Ad libitum food was displaced in the home cage the day before the surgery and until the end of the recovery period. The cannula cover was removed by cutting the part of the metal tube between the cannula and the cement the day before the first injection.

We infused 0.5 μl of drug solution intracerebrally (i.c.) at a rate of 0.15 μl/min through a 28 G injection cannula that protruded 1 mm beyond the tip of the guide cannula. The injection cannula was kept in place for additional 2 min to allow drug diffusion. MPEP (40 μM) or vehicle (saline) (0.5 μL/side each) was infused 30 min before the training of the 12th, 14th and 16th VI-60 session.

### In vivo DHPG brain infusion

Mice were subjected to short training regimes up to the first VI-60 session, 24 h after which animals were anesthetized with isoflurane. For infusions in the DLS, borosilicate capillaries [Ø 1/0.78 mm (external/internal), Warner Instruments] were used. The capillaries were pulled with a vertical puller to obtain a long, thin tip (0.5–1 mm long, Ø 50 μm), and connected through a tube to a 10 μl Hamilton syringe. The whole system was filled with saline, and a 1 μl bubble was left at the end of the capillary tip. The capillary was then loaded with the 100 μM DHPG solution obtained by diluting the DHPG (Tocris) from a stock concentration of 10 mM. In case of vehicle infusion, no DHPG was included, and only water was loaded. A 0.5 μl of DHPG solution (100 μM) was infused intracerebrally (i.c.) at a rate of 0.15 μl/min, and the capillary was left in place to favor diffusion for 5 min and then slowly retracted in 2–3 min. The opposite hemisphere was infused with vehicle (water) and used as an internal control to avoid the confounding factor of mechanical stress during surgery and infusion. DHPG- and vehicle-hemisphere infusions were randomized between every animal. Ten minutes after infusion, the animal was sacrificed and brain samples were processed using the normal procedure described under Western Blotting.

### Brain slice preparation

Brain slices containing the striatum and cortex were prepared as described [48,54]. Mice were anesthetized with isofluorane and decapitated, and their brains were rapidly transferred to ice-cold dissecting aCSF containing 110 mM Choline-Cl, 2.5 mM KCl, 1.25 mM NaH_2_PO_4_, 7 mM MgCl_2_6H_2_O, 0.5 mM CaCl_2_, 25 mM NaHCO_3_, 25 mM D-glucose, 11.6 mM ascorbic acid, and saturated with 95% O_2_ and 5% CO_2_. Horizontal corticostriatal slices (270 μm thick) were cut in the dissecting aCSF using a Vibrotome 1000S slicer (Leica, Italy), then transferred to normal aCSF containing 115 mM NaCl, 3.5 mM KCl, 1.2 mM NaH_2_PO_4_, 1.3 mM MgCl_2_6H_2_O, 2 mM CaCl_2_, 25 mM NaHCO_3_, and 25 mM D-glucose, and aerated with 95% O_2_ and 5% CO_2_. Following 20 min of incubation at 32 °C, slices were kept at RT. During experiments, slices were continuously superfused with aCFS at a rate of 2 ml/min at 28 °C.

### Electrophysiology

#### Extracellular field recordings.

Extracellular field recordings of glutamate-driven population spikes (PS) were obtained in the dorsolateral striatum (DLS) or in the dorsomedial striatum (DMS) using glass micropipettes filled with 3 M NaCl, as described [20]. Stimuli were delivered via a Constant Voltage Isolated Stimulator (Digitimer, Welwyn Garden City, UK) through a bipolar twisted tungsten electrode placed in the proximity of the white matter overlaying the DLS and acquired every 30 s. Data were amplified and filtered (low filter 10 Hz, high filter 3 kHz) by a DAM 80 AC Differential Amplifier (World Precision Instruments, Sarasota, FL), and digitized at 10 KHz by a Digidata 1,322 (Molecular Devices, Foster City, CA). Depression of PS amplitude was induced by (RS)-3,5-DHPG application (15 min). Recordings were performed in the presence of the GABA_A_ receptor antagonist gabazine (10 µM). PS amplitudes were measured using Minianalysis program (Synaptosoft, Decatur, GA). Comparison of presynaptic fiber volley (N1 component) before and after (RS)-3,5-DHPG was used as an internal control to test that changes in PS amplitude were not due to differences in axonal activity or slice health. Recordings in which volley amplitude changed by more than 20% were discarded. LTD plots were generated by averaging the peak amplitude of individual EPSPs in 2-min bins.

#### Patch-clamp recordings.

Whole-cell recordings were made under direct IR-DIC (infrared-differential interference contrast) visualization of neurons in the DLS, which were identified as striatal projection neurons (SPNs) based on morphological and electrical properties. SPNs were filled with Neurobiotin (0.5 mg/ml) during recordings and subsequently processed for immunostaining against A2A receptor for iSPNs and substance P for dSPNs [48]. Current clamp experiments were performed by using borosilicate patch pipettes (4–6 MΩ) filled with a solution containing 135 mM KMeSO_4_, 10 mM KCl, 10 mM HEPES, 1 mM MgCl_2_, 2 mM Na_2_-ATP, 0.4 mM Na_3_-GTP (pH 7.2–7.3, 280–290 mOsm/kg). SPNs were clamped at a holding membrane potential of −80 mV. Excitatory postsynaptic potentials (EPSPs) were evoked in the presence of the GABA_A_ receptor antagonist gabazine (10 μM) by cortical stimulation from the somatosensory cortex layer 5 by using a concentric bipolar electrode (80 µsc–200 µsc, 0.9 mA–1.6 mA CBAPB75, FHC, Bowdoin, ME) connected to a constant-current isolation unit (Digitimer LTD, Model DS3) and acquired every 10 s. The synaptic activation of the eCB-signaling was induced using a spike-timing-dependent plasticity (STDP) protocol consisting of 20 bouts of EPSPs paired with back-propagating action potentials (bAPs), delivered 10 s apart. Each bout consisted of five bursts (120 ms apart) each composed of three bAPs at 50 Hz followed by one EPSP (negative timing) [20]. The onset of the EPSPs followed the peak of the last postsynaptic action potential in the burst by 10 ms (Δ*t* = 10 ms). During plasticity induction, postsynaptic neurons were depolarized from −80 mV to −70 mV. Signals were sampled at 20 kHz and filtered to 10 kHz. The occurrence and magnitude of synaptic plasticity in each experiment was evaluated by comparing the normalized EPSP amplitudes from the last 5 min of baseline recordings with the values between 25 and 35 min after STDP. LTD plots were generated by averaging the peak amplitude of individual EPSPs in 2-min bins. The coefficient of variation (CV) for EPSP was calculated by the ratio of the standard deviation (sd) and the mean EPSP amplitude [48,54,96].

Voltage clamp experiments were performed on SPNs using borosilicate patch pipettes (3–4 MΩ) filled with a solution containing 130 mM CeMeSO_3_, 5 mM CsCl, 5 mM NaCl, 10 mM HEPES, 0.1 mM EGTA, 2 mM MgCl_2_, 0.05 mM CaCl_2_, 2 mM Na_2_-ATP, and 0.4 mM Na_3_-GTP (pH 7.2–7.3, 280–290 mOsm/kg). Spontaneous AMPA-mediated miniature excitatory postsynaptic currents (mEPSCs) were recorded in gap-free mode for 10 min. SPNs were clamped at a holding membrane potential of –70 mV, by adding 10 µM gabazine and 0.5 µM TTX to the recording solution. mEPSC frequency was analyzed offline using Mini analysis software (Synaptosoft, Decatur, GA). Automated detection of mEPSCs was verified by visual inspection, with the experimenter blind to the experimental condition during analysis. NMDA receptor-mediated EPSCs were pharmacologically isolated in aCSF containing 10 µM gabazine and 20 μM NBQX disodium salt to block AMPA-mediated currents. SPNs were voltage-clamped at +40 mV and NMDA EPSCs were evoked by cortical stimulation from the somatosensory cortex layer 5. To study the Ro25–6981-sensitive component in total NMDA EPSCs, Ro25–6981 was bath-applied for 25 min, during which NMDA EPSCs were evoked every minute after obtaining a stable baseline of 5 min. Access resistance was monitored throughout the experiment. Signals were sampled at 10 kHz and filtered at 2.4 kHz. For both current and voltage-clamp experiments, only cells with a stable resting membrane potential ≤−78 mV were included in the analysis. Series resistance (range 15–25 MΩ) was monitored at regular intervals throughout the recording and presented minimal variations (≤20%) in the analyzed cells. Data are reported without corrections for liquid junction potentials. Data were acquired using a Multiclamp 700B amplifier controlled by pClamp 10 software (Molecular Device), with a Digidata 1,322 (Molecular Device).

### Immunofluorescence

#### Identification of D2 SPN and D1 SPN.

During electrophysiology experiments, SPNs were filled with Neurobiotin (0.5 mg/ml) dissolved in the intracellular solution, as described [48]. At the end of the recordings, the slices were fixed with 4% paraformaldehyde in PB overnight at 4 °C. After antigen retrieval in a solution of sodium citrate (50 mM, for 30 min at 80 °C), the slices were incubated in primary antibodies for 24 h at room temperature, followed by 48 h at 4 °C. Rabbit polyclonal antibody for A2AR (1:250, Enzo Biosciences) and rat monoclonal antibody for substance P (1:200, Millipore) were diluted in 0.1 M PB containing 0.3% (v/v) Triton X-100 and 0.02% NaN_3_. Next, sections were incubated 4 h in a diluted solution of Alexa 568-conjugated streptavidin (1:5000; Invitrogen), followed by 48 h of incubation at 4 °C with Alexa 647- and Alexa 488-conjugated secondary antibodies (Invitrogen). Slices were then mounted on glass slides with ProLong Gold Antifade reagent (Invitrogen) and covered by coverslips. Images were acquired with an inverted confocal microscope (TCS SP5 AOBS TANDEM, Leica).

### Western blotting

Animals were anesthetized with isofluorane and transcardially perfused with ice-cold PBS containing phosphatase inhibitor cocktail 2 at 1:100 (Sigma), phosphatase inhibitor cocktail 3 at 1:100 (Sigma), 10 mM NaF (final), and 1 mM sodium ortovanadate. Dorsolateral and dorsomedial parts of the striatum were dissected and brain samples were extracted before or 10 min after the second VI-60 session and then processed. Brains were placed in a slicing chamber maintained on ice at 4 °C, and striatal slices were obtained by placing razor blades in the slicing chamber rails. Razor blades containing slices were then placed on dry ice. Razor blades were then taken out from the dry ice, and placed onto a cold surface (dry ice wrapped in aluminum foil and paper towels). Dissected tissues were then placed into Eppendorf tubes apposed on dry ice. The samples were then conserved at −80 °C.

Brain tissue samples were homogenized in 100 µl lysis buffer with 2% SDS, 5 mM EGTA pH 8, 20 mM HEPES, plus threonine/serine phosphatase inhibitors; Cocktail 3 (Sigma) at 1:100, Cocktail 2 (Sigma) at 1:100, 1× protease inhibitor cocktail (Roche). Lysates were then heated 3 min at 99 °C and centrifugated 15 min at 13,000 RPM at 24 °C. After centrifugation, 95 µl of the supernatant was collected and placed in fresh Eppendorf tubes, and 5 µL was kept for BCA processing. A 5× Sample buffer (SB5×) was added to the remaining supernatant, and all cytosolic extract was then aliquoted and stored at −80 °C.

Protein content for each sample was determined using the Pierce BCA protein assay kit (Thermo Scientific). Briefly, 200 µL working reagent solution (Thermo Scientific) was added per 5× diluted samples duplicates, analyzed samples and standard samples were incubated 30 min at 37 °C, and duplicates were then processed by a VICTOR^3^V multilabel counter (Perkin Elmer) and analyzed by the software Wallac 1420.

Aliquots containing 20 µg of protein were subjected to sodium dodecyl sulfate polyacrylamide gel electrophoresis (SDS-PAGE); all aliquots were normalized with 1× sample buffer. Proteins were transferred onto nitrocellulose membranes. After transfer, membranes were blocked in either 5% milk plus 0.1% TBS-T, or 5% BSA plus 0.1% TBS-T. Membranes were next incubated with primary antibodies against phospho-Akt (Ser 473; 1/5000 dilution) (Cell Signaling) or GluN2B (Neuromab) at 4 °C overnight. Each primary antibody incubation was followed by incubation for 1 h with a secondary horseradish peroxidase-conjugated goat antibody diluted at 1:15,000 in blocking buffer. Blots were developed using Amersham ECL Western Blotting detection reagent (GE Healthcare) and exposed to Amersham Hyperfilm ECL (GE Healthcare). Multiple exposures of each membrane were taken to ensure the linearity of the immunoreactive bands. For reuse of membranes that had been Western blotted, a mild stripping procedure was performed using stripping buffer (2% SDS, 6.5% Tris pH 7; 0.7% β-mercapto-EtOH). The stripped blots were blocked and incubated with an antibody against total levels of Akt at 1/5000 dilution (Cell Signaling), followed by incubation with an antibody against calnexin at 1/1000 dilution (EnzoLifeSciences) as a loading control. Densitometric analysis of phospho- and total immunoreactivity for each protein was conducted using Image QTL software (GE Healthcare). Phosphorylated immunoreactivity was normalized to total protein immunoreactivity for each of the proteins assessed. When phosphorylation was not analyzed, total immunoreactivity was normalized to loading control proteins. Each quantification was then normalized to its respective control condition; dorsomedial or dorsolateral short-trained samples for omission and trained groups.

### Crude membrane fraction purification

DLS samples were homogenized at 4 °C in an ice-cold buffer containing 0.32 M sucrose, 1 mM HEPES, 1 mM NaF, 0.1 mM phenylmethylsulfonyl fluoride (PMSF), and 1 mM MgCl_2_ in the presence of protease inhibitors (Complete, GE Healthcare) and phosphatase inhibitors (PhosSTOP, Roche Diagnostics GmbH), using a glass–Teflon homogenizer. Homogenates were then centrifuged at 800 × *g* for 5 min at 4 °C, to remove nuclear contamination and white matter. The supernatant was collected and centrifuged at 13,000 × *g* for 15 min at 4 °C. The resulting pellet (the P2 crude membrane fraction) was resuspended in a buffer containing 20 mM HEPES and Complete. Protein content of the samples was quantified by using Bio-Rad protein assay. After measuring protein concentration, the same protein amount was loaded onto a 7% SDS-PAGE gel and revealed by Western Blotting with the following antibodies: GluN2B-P1472 (Calbiochem), GluN2B (Neuromab), and Tubulin (Sigma).

### Statistics

Appropriate parametric statistics were used to test hypotheses unless data did not meet the assumptions of the intended parametric test (normality test). In this case, appropriate non-parametric tests were used. Power analysis specifications to estimate sample size were: power = 0.8, alpha = 0.05, two-tailed, and an effect size that is 50% greater than previously observed standard deviations. Data were analyzed by two-way repeated measure ANOVA (RM2WA) or one-way repeated measure ANOVA (RM1WA) for comparisons within a group, and one-way ANOVA (1WA) for between-group comparisons (GraphPad Prism 9 software). A mixed-effects two-way ANOVA (MDA) was used to analyze experiments with between-subjects (short- and overtraining) and within-subjects variables (post-training positive A–O versus negative A–O or valued versus devalued conditions). Corrected post-hoc tests (Tukey or Sidak as indicated) were performed only when the ANOVA yielded a significant main or interaction effect. Two groups were tested for statistical significance using the independent samples *t* test, the paired samples *t* test, or equivalent non-parametric tests (GraphPad Prism 9 software). Statistical details of experiments are shown in the results, figure legends, and in the S1 Table.

## Supporting information

S1 Fig Related to Fig 1. **(A)** (Top) Schematic depicts the behavioral paradigms. (Bottom) Magazine entry (ME) rates (left) and inactive nose-poke (INP) rates (right) during training in the two experimental groups (T, *n* = 9; T_S, *n* = 9; ME/min, session: *F*_5,80_ = 10, ****p < 0.0001, group: *F*_1,16_ = 1, *p* = 0.3, interaction: *F*_5,80_ = 0.2, *p* = 0.9; INP/min, session: *F*_5,80_ = 1.3, *p* = 0.3, group: *F*_1,16_ = 1.4, *p* = 0.3, interaction: *F*_5,80_ = 0.4, *p* = 0.9). Data are presented as mean ± SEM. **(B)** (Top) Schematic depicts the behavioral regimes and in vivo DLS infusions. (Bottom) Active nose-pokes (ANP) (left), magazine entry (ME) (middle) and inactive nose-poke (INP) (right) rates in the different experimental groups (T_, *n* = 15; T_S Veh, *n* = 13; T_S MPEP *n* = 8; ANP/min: *F*_5,165 _= 52.10, ****p < 0.0001, group: *F*_2,33 _= 1.813, *p* = 0.2, interaction: *F*_10,165 _= 1.539, *p* = 0.1; ME/min, session: *F*_5,165 _= 22.82, ****p < 0.0001, group: *F*_2,33 _= 0.6297, *p* = 0.5, interaction: *F*_10,165 _= 1.655, *p* = 0.1; INP/min, session: *F*_5,165 _= 1.996, *p* = 0.1, group: *F*_2,33 _= 1.032, *p* = 0.4, interaction: *F*_10,165 _= 1.243, *p* = 0.3). *Inset*, ANP/min in T_S Veh and T_S MPEP mice, 30 min after DLS infusion (Mann–Whitney test, *p* = 0.12). Data are presented as mean ± SEM. **(C)** Representative western blots of pAkt, Akt, and Calnexin in the DLS of T_, T_S Veh, T_S MPEP mice. Bar graphs are expression level ratios (relative to T_) of pAkt/Akt (T__pAkt/Akt_: 1.00 ± 0.1265, *n* = 15; T_S Veh _pAkt/Akt_: 1.81 ± 0.2446, *n* = 13; T_S MPEP_pAkt/Akt_: 0.7443 ± 0.1597, *n* = 8. T__pAkt/Akt_ versus T_S Veh_pAkt/Akt_, Dunn’s test, *p = 0.044; T__pAkt/Akt_ versus T_S MPEP_pAkt/Akt_, Dunn’s test, *p* > 0.99; T_S Veh_pAkt/Akt_ versus T_S MPEP_pAkt/Akt_, Dunn’s test, *p = 0.011). **(D)** Schematic of short- and overtraining followed by the post-training omission or devaluation procedures. **(E)** Averaged time courses of magazine entry (ME) rates (left) and inactive nose-poke (INP) rates (right) during training (Sh, *n* = 19; Ov, *n* = 16; ME/min, session: *F*_8,264_ = 15, ****p < 0.0001, group: *F*_1,33_ = 0.008, *p* = 0.9, interaction: *F*_8,264_ = 1.7, *p* = 0.09; IN/min, session: *F*_8,264_ = 1.4, *p* = 0.2, group: *F*_1,33_ = 0.07, *p* = 0.8, interaction: *F*_8,264_ = 1.5, *p* = 0.15). **(F)** Post-training omission procedure in short- and overtrained mice (Sh *n* = 10, Ov *n* = 8). (Left) INP rates (A–O contingency: *F*_1,16_ = 0.001, *p* = 1, group: *F*_1,16_ = 0.14, *p* = 0.7, A–O contingency × group interaction: *F*_1,16_ = 0.28, *p* = 0.6; Sh: positive A–O: 0.38 ± 0.19, negative A–O, 0.35 ± 0.20, Sidak: *p* = 0.9; Ov: positive A–O 0.27 ± 0.06, negative A–O: 0.3 ± 0.1, Sidak: *p* = 0.9). (Right) Number of obtained reinforcers (pellets; g) in the positive- and negative A–O contingency sessions (Sh_Positive A–O: 0.35 ± 0.007, Ov_Positive A–O: 0.35 ± 0.02, Sidak: *p* = 1; Sh_Negative A–O: 0.49 ± 0.03, Ov_Negative A–O: 0.23 ± 0.07, Sidak: ****p < 0.0001). **(G)** Post-training devaluation procedure in short- and overtrained mice (Sh *n* = 9, Ov *n* = 8). (Left) INP rates in the valued and devalued conditions (Sh, valued: 0.31 ± 0.09; devalued: 0.27 ± 0.08; Ov, valued: 0.58 ± 0.18; devalued: 0.68 ± 0.16; condition: *F*_1,15_ = 0.13, *p* = 0.7, group: *F*_1,15_ = 4.07, *p* = 0.06, condition × group interaction: *F*_1,15_ = 0.88, *p* = 0.4; Sh, valued versus devalued, Sidak *p* = 0.89; Ov, valued versus Ov, devalued, Sidak *p* = 0.62; (Right) Pellet consumption (g) in the valued and devalued conditions (Sh, valued: 0.51 ± 0.07; devalued: 0.8 ± 0.08; Ov, valued: 0.69 ± 0.13; devalued: 0.65 ± 0.09; condition: *F*_1,15_ = 2.3, *p* = 0.15, group: *F*_1,15_ = 0.006, *p* = 0.94, condition × group interaction: *F*_1,15_ = 3.9, *p* = 0.07; Sh, valued versus devalued, Sidak *p = 0.04; Ov, valued versus devalued, Sidak *p* = 0.94). Data are presented as: mean ± SEM (**E**; **F–G** right); values are the minimum, mean (bar inside the box), and maximum (**F–G** left). Data set are available at the following link: https://doi.org/10.48557/VCAWUD.
(PDF)

S2 Fig Related to Fig 2. **(A)** Schematic of behavioral training paradigms. **(B)** ANP/min (session: *F*_8,1,120_ = 301.4, ****p < 0.0001; group: *F*_3,140_ = 1.353, *p* = 0.2; interaction: *F*_24,1,120_ = 1.735, *p = 0.015), ME (session: *F*_8,1,120_ = 39.06, ****p < 0.0001; group: *F*_3,140_ = 1.121, *p* = 0.34; interaction, *F*_24,1,120_ = 2.414, ***p = 0.0002) and INP/min (session: *F*_8,1,120_ = 2.649, **p = 0.007; group:: *F*_3,140_ = 1.517, *p* = 0.21; *F*_24,1,120_ = 1.416, *p* = 0.09) during instrumental learning in the different experimental groups (Sh, *n* = 29; Sh ΔAO, *n* = 47; Ov, *n* = 23; Ov ΔAO, *n* = 45). Symbols represent the positive A–O performance for mice not undergoing omission procedure. **(C)** Post-training omission procedure. (Left) Comparison of ANP rates between positive and negative A–O contingency in both short- and overtrained mice (A–O contingency: *F*_1,90_ = 58.45, ****p < 0.0001, group: *F*_1,90_ = 4.837, **p = 0.0304, A–O contingency × group interaction: *F*_1,90_ = 21.33, ****p < 0.0001; Sh *n* = 47, positive A–O: 15.33 ± 1.05, negative A–O: 8.35 ± 0.80, Sidak ****p < 0.0001; Ov *n* = 45, positive A–O: 15.50 ± 1.002, negative A–O 13.90 ± 0.79; Sidak *p* = 0.07). (Right) Time courses of ANP ratio that indicate a main group effect (*F*_5,450_ = 11.11, ****p < 0.0001; *F*_1,90_ = 27.02, ****p < 0.0001; interaction: *F*_5,450_ = 3.457, **p = 0.0045. **(D–E)** Comparison of INP rates **(D)** and obtained reinforcers **(E)** between positive and negative A–O contingency, in both short- (*n* = 47) and overtrained (*n* = 45) mice (INP/min; A–O contingency: *F*_1,90_ = 0.119, *p* = 0.73, group: *F*_1,90_ = 0.047, *p* = 0.82, A–O contingency × group interaction: *F*_1,90_ = 0.84, *p* = 0.36; Sh, positive A–O: 0.34 ± 0.09, negative A–O: 0.30 ± 0.07, Sidak: *p* = 0.60; Ov, positive A–O: 0.29 ± 0.045, negative A–O 0.31 ± 0.06; Sidak: *p* = 0.90; Reinforcers, A–O contingency: *F*_1,90_ = 14.7, ***p = 0.0002, group: *F*_1,90_ = 25.21, ****p < 0.0001, A–O contingency × group interaction:, *F*_1,90_ = 20.9, ****p < 0.0001; Sh_Positive A–O: 0.38 ± 0.006, Ov_Positive A–O: 0.39 ± 0.003; Sidak *p* = 0.83; Sh_Negative A–O: 0.39 ± 0.03, Ov_Negative A–O 0.22 ± 0.02; Sidak ****p < 0.0001). **(F)** Depression of PS responses in the DMS, following bath application of DHPG (100 μm) in Sh_ and Ov_mice (Sh_: slices *n* = 7, mice *n* = 4; *F*_6,22 _= 10.0, ****p < 0.0001, Tukey’s *p < 0.05; Ov_: slices *n* = 7, mice *n* = 4; *F*_6,22 _= 27.84, ****p < 0.0001, Tukey’s *p < 0.05; Sh_ *versus* Ov_, Unpaired *t* test, *p* = 0.52, *t* = 0.67, *df* = 12). *Insets*, averaged recordings from slices before (black line) and after DHPG application (red line). Scale bars: 0.1 mV/1 ms. **(G)** Depression of PS responses in the DMS, following bath application of DHPG (100 μm) in Sh_Δ A–O and Ov_Δ A–O mice (Sh_Δ A–O: slices *n* = 6, mice *n* = 4; *F*_5,22 _= 9.46, ****p < 0.0001, Tukey’s *p < 0.05; Ov_Δ A–O: slices *n* = 8, mice *n* = 4; *F*_7,22 _= 18.96, ****p < 0.0001, Tukey’s *p < 0.05; Sh_Δ A–O *versus* Ov_ Δ A–O, Unpaired *t* test, *p* = 0.86, *t* = 0.18, *df* = 12). *Insets*, averaged recordings from slices before (black line) and after DHPG application (red line). Scale bars: 0.1 mV/1 ms. Data set are available at the following link: https://doi.org/10.48557/VCAWUD.
(PDF)

S3 Fig Related to Fig 3. **(A)** Schematic of the behavioral regimes followed by ex vivo electrophysiology. **(B)** The CB1 antagonist AM251 (4 μM) prevented t-LTD at cortico-iSPN synapses in Sh_Δ A–O mice (cells *n* = 6, mice *n* = 3; *F*_5,22_ = 0.4, *p* = 0.7; iSPN_Sh_Δ A–O versus iSPN_Sh_Δ A–O + AM251, Mann–Whitney test, **p = 0.004, *U* = 1). Solid black line (average) is the time course from Fig 3C, reported here for comparison. Data are presented as time courses (mean ± SEM) of normalized EPSP amplitudes and normalized Rinp. Scatterplot summarizes the ratios of synaptic responses after (a) and before (b) the STDP. Insets represent superimposed averaged recordings (10 traces) before (black line) and after (green line) the delivery of the STDP protocol (green vertical bar), and the proposed signaling elements targeted by the defined antagonist. **(C)** (Top) Representative traces for mEPSC recorded at iSPN synapses in Ov_ (24 h later) compared to Ov_Δ Α–Ο (30 min later) mice. (Bottom) Scatterplot comparing mEPSC frequency (Hz) and amplitude (pA) in the two mouse groups (iSPN_Ov_: cells *n* = 7, mice = 4; frequency 1.82 ± 0.27 Hz, amplitude 10.85 ± 0.5 pA; iSPN_Ov_Δ A–O: cells *n* = 6, mice = 4; frequency (Hz) 1.46 ± 0.24 Hz, amplitude 9.39 ± 0.90 pA; iSPN_ Ov_ *versus* iSPN_Ov_Δ A–O: frequency, Mann–Whitney test, *p* = 0.23, *U* = 12; amplitude: Mann–Whitney test, *p* = 0.2, *U* = 12). **(D)** (I) Comparison of CV^−2^ of evoked EPSPs in iSPN_Ov_(cells *n* = 6, mice *n* = 5, 41.01 ± 12.98) and iSPN_Ov_Δ A–O (cells *n* = 8, mice *n* = 7, 37.33 ± 12.20, iSPN_ Ov_ *versus* iSPN_Ov_Δ A–O, Mann–Whitney test, *p* > 0.99, *U* = 24). CV^−2^ calculations were based on 60 sweeps (10 min recordings). Data are presented as mean ± SEM. Data set are available at the following link: https://doi.org/10.48557/VCAWUD.
(PDF)

S4 Fig Related to Fig 4. **(A)** Schematic of the behavioral paradigms and in vivo pharmacological manipulation. **(B)** ME rates (left) and INP rates (right) during training in Veh_Ov, MPEP_Ov, and in the control Sh group (Sh, *n* = 8, Veh_Ov, *n* = 26, MPEP_Ov, *n* = 25; ME/min, session: *F*_8,448_ = 22, ****p < 0.0001; group: *F*_2,56_ = 3, *p* = 0.08; interaction: *F*_8,448_ = 3, ***p = 0.0005; INP/min, session: *F*_8,448_ = 4, ***p = 0.0004; group: *F*_2,56_ = 1.2, *p* = 0.3; interaction: *F*_8,448_ = 1.6, *p* = 0.06). **(C)** Comparison of INP rates (left) and obtained reinforcers (right) between positive and negative A–O contingency in the different experimental groups (Sh, *n* = 8, Veh_Ov, *n* = 19, MPEP_Ov, *n* = 15; INP/min; A–O contingency: *F*_1,39_ = 0.001, *p* = 0.97; group: *F*_2,39_ = 0.85, *p* = 0.43; A–O contingency × group interaction, *F*_2,39_ = 0.45, *p* = 0.64; Sh, positive A–O: 0.33 ± 0.07, negative A–O: 0.25 ± 0.6, Sidak: *p* = 0.87; Veh_Ov, positive A–O: 0.39 ± 0.06, negative A–O: 0.44 ± 0.08, Sidak: *p* = 0.89; MPEP_Ov, positive A–O: 0.32 ± 0.9, negative A–O: 0.35 ± 07, Sidak: *p* = 0.98; reinforcers; Sh_Positive A–O: 0.39 ± 0.006, Veh_Ov_Positive A–O: 0.39 ± 0.008; MPEP_Ov_Positive: 0.40 ± 0.009; Sh_Positive A–O versus Veh_Ov_Positive A–O, Sidak *p* > 0.99, Sh_Positive A–O versus MPEP_Ov_Positive A–O, Sidak *p* = 0.98, Veh_Ov_Positive A–O versus MPEP_Ov_Positive A–O, Sidak *p* = 0.96; Sh_Negative A–O, 0.29 ± 0.05, Veh_Ov_Negative A–O, 0.15 ± 0.02, MPEP_Ov_Negative A–O, 0.31 ± 0.04; Sh_Negative A–O versus Veh_Ov_Negative A–O, Sidak ***p = 0.0008, Sh_Negative A–O versus MPEP_Ov_Negative A–O, Sidak *p* = 0.93,Veh_Ov_Negative A–O versus MPEP_Ov_Negative A–O, Sidak, ****p < 0.0001). **(D)** Post-training devaluation procedure in the vehicle and MPEP-treated overtrained mice (Veh_Ov, *n* = 7, MPEP_Ov, *n* = 10). (Left) ANP rates (Veh_Ov_Valued, 4.11 ± 1.01, Veh_Ov_Devalued, 4.70 ± 1.12, MPEP_Ov_Valued, 3.8 ± 0.87, MPEP_Ov_Devalued, 3.06 ± 0.58; Veh_Ov_Valued *versus* Veh_Ov_Devalued, Sidak *p* = 0.91, MPEP_Ov_Valued *versus* MPEP_Ov_Devalued; Sidak *p* = 0.76). (Middle) INP rates (Veh_Ov_Valued, 0.63 ± 0.12; Veh_Ov_Devalued, 0.45 ± 0.11; MPEP_Ov_Valued, 0.46 ± 0.13; MPEP_Ov_Devalued, 0.28 ± 0.04; condition: *F*_1,15_ = 1.8, *p* = 0.2, group: *F*_1,15_ = 2.3, *p* = 0.15, condition × group interaction, *F*_1,15_ = 0.001, *p* = 0.97; Veh_Ov_Valued *versus* Veh_Ov_Devalued, Sidak *p* = 0.65, MPEP_Ov_Valued *versus* MPEP_Ov_Devalued, Sidak *p* = 0.51). (Right) Pellet consumption (g), (Veh_Ov_Valued, 0.56 ± 0.09; Veh_Ov_Devalued, 0.51 ± 0.05; MPEP_Ov_Valued, 0.64 ± 0.08; MPEP_Ov_Devalued, 0.67 ± 0.08; condition: *F*_1,15_ = 0.02, *p* = 0.89, group: *F*_1,15_ = 1.3, *p* = 0.26, condition × group interaction, *F*_1,15_ = 0.48, *p* = 0.5, Veh_Ov_Valued *versus* Veh_Ov_Devalued, Sidak *p* = 0.84, MPEP_Ov_Valued *versus* MPEP_Ov_Devalued, Sidak *p* = 0.89. (**B–D**) Data are presented as mean ± SEM. Data set are available at the following link: https://doi.org/10.48557/VCAWUD.
(PDF)

S5 Fig Related to Fig 5**. (A)** The L-type VGCC blocker nimodipine (10 μM) blocked dSPN t-LTD gated upon co-application of MPEP and Ro-256981 (Ro) during the negative STDP (dSPN_Naïve + MPEP + Ro + Nimodipine, cells *n* = 6, mice *n* = 6, *F*_5,22_ = 1, *p* = 0.4; dSPN_Naïve + MPEP + Ro versus dSPN_Naïve + MPEP + Ro + Nimodipine, Mann–Whitney test, **p = 0.008, *U* = 4). Solid black line (average) is the time course from Fig 5A, reported here for comparison. **(B)** Application of either MPEP or Ro alone during negative STDP failed to induce t-LTD in dSPNs (dSPN_Naïve + MPEP, cells *n* = 5, mice *n* = 4; *F*_4,22 _= 0.6, *p* = 0.6; dSPN_Naïve + Ro, cells *n* = 5, mice *n* = 4; *F*_4,22 _= 0.97, *p* = 0.4; group comparison; *F*_2,12 _= 1.3, *p* = 0.3). **(A–B)** Solid black lines (average) are the time course of MPEP + Ro **(A)** and control **(B)** conditions from Fig 5A, reported here for comparison. Data are time courses (mean ± SEM) of normalized EPSP amplitudes and normalized Rinp. Scatterplots are ratios of synaptic responses after (a) and before (b) the STDP. Insets represent superimposed averaged recordings (10 traces) before (black line) and after (green line) the delivery of the STDP protocol (green vertical bar), and the proposed signaling elements targeted by the defined drugs. Data set are available at the following link: https://doi.org/10.48557/VCAWUD.
(PDF)

S1 Table Statistical table. (XLSX)

S1 Raw Images Original blot images for 1C, 1F, 2B, 2C, 2E, 2F, 2H, 4D, 5D, 5F, S1C. (PDF)

## Abbreviations

A2AR: A2A receptor
AIP: Autocamtide-2-Related Inhibitory Peptide
ANP: active nose-poke
A–O: action–outcome
bAPs: back-propagating action potentials
CaMKII: calcium/calmodulin-dependent protein kinase II
CB1R: cannabinoid receptor type 1
CRF: continuous reinforcement
CV: coefficient of variation
DGL: diacylglycerol lipase
DLS: dorsolateral striatum
DMS: dorsomedial striatum
D1R: D1 receptors
D2R: D2 receptors
eCB: endocannabinoid
EPSP: excitatory postsynaptic potentials
GBZ: Gabazine
INP: inactive nose-pokes
LTD: long-term synaptic depression
ME: magazine entries
MPEP: Methyl-6-(phenylethynyl)pyridine hydrochloride
NMDAR: NMDA receptor
Ov_: overtrained
PKC: protein kinase C
PMSF: phenylmethylsulfonyl fluoride
PS: postsynaptic
SEM: standard error of the mean
Sh_: short-trained
SP: substance P
SPN: striatal projection neurons
STDP: spike-timing-dependent plasticity
tLTD: timing-dependent synaptic depression
TTx: tetrodotoxin
Veh: vehicle
VI: variable interval.
