# Supplementary figures and images for: Behavioral inflexibility through overtraining is mediated by reduced mGluR1/5 signaling capacity in the dorsolateral striatum
Source: PLoS Biol. 2025 Jul 29;23(7):e3003288. doi: 10.1371/journal.pbio.3003288 (PMC12327642; doi:10.1371/journal.pbio.3003288)

S1 Fig

A

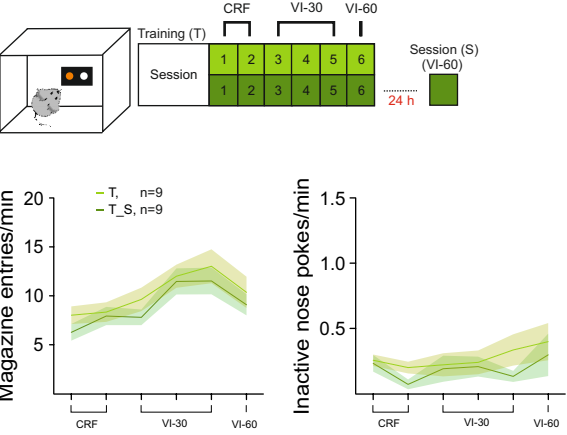

B

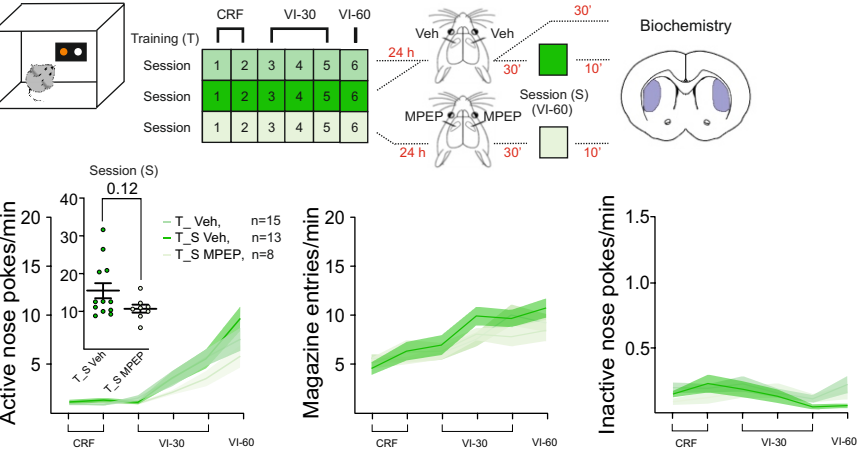

D

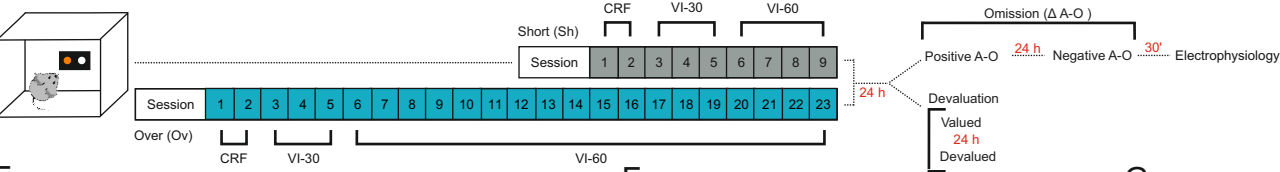

E

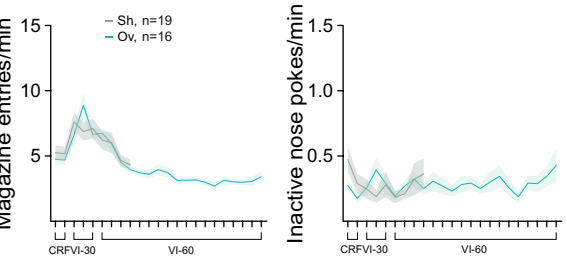

F

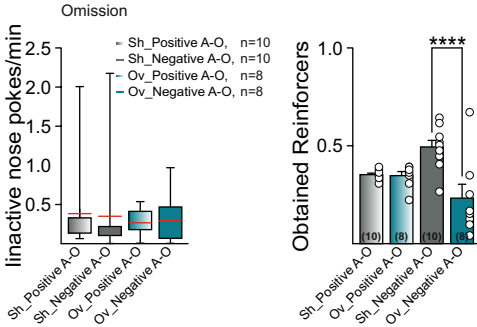

G

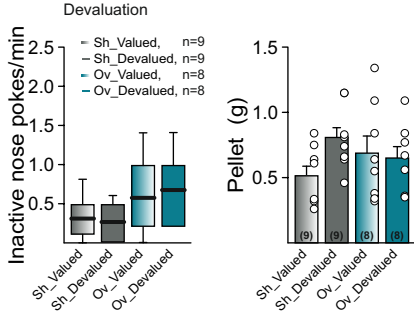

C

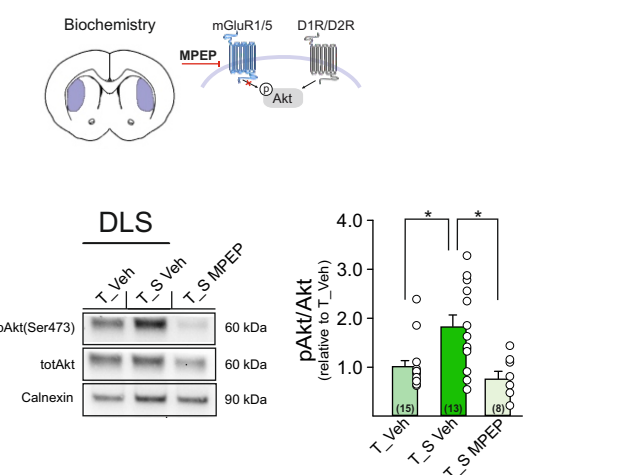

Supplement: S1 Fig — (A) (Top) Schematic depicts the behavioral paradigms. (Bottom) Magazine entry (ME) rates (left) and inactive nose-poke (INP) rates (right) during training in the two experimental groups (T, n = 9; T_S, n = 9; ME/min, session: F5,80 = 10, ****p < 0.0001, group: F1,16 = 1, p = 0.3, interaction: F5,80 = 0.2, p = 0.9; INP/min, session: F5,80 = 1.3, p = 0.3, group: F1,16 = 1.4, p = 0.3, interaction: F5,80 = 0.4, p = 0.9). Data are presented as mean ± SEM. (B) (Top) Schematic depicts the behavioral regimes and in vivo DLS infusions. (Bottom) Active nose-pokes (ANP) (left), magazine entry (ME) (middle) and inactive nose-poke (INP) (right) rates in the different experimental groups (T_, n = 15; T_S Veh, n = 13; T_S MPEP n = 8; ANP/min: F5,165 = 52.10, ****p < 0.0001, group: F2,33 = 1.813, p = 0.2, interaction: F10,165 = 1.539, p = 0.1; ME/min, session: F5,165 = 22.82, ****p < 0.0001, group: F2,33 = 0.6297, p = 0.5, interaction: F10,165 = 1.655, p = 0.1; INP/min, session: F5,165 = 1.996, p = 0.1, group: F2,33 = 1.032, p = 0.4, interaction: F10,165 = 1.243, p = 0.3). Inset, ANP/min in T_S Veh and T_S MPEP mice, 30 min after DLS infusion (Mann–Whitney test, p = 0.12). Data are presented as mean ± SEM. (C) Representative western blots of pAkt, Akt, and Calnexin in the DLS of T_, T_S Veh, T_S MPEP mice. Bar graphs are expression level ratios (relative to T_) of pAkt/Akt (T_pAkt/Akt: 1.00 ± 0.1265, n = 15; T_S Veh pAkt/Akt: 1.81 ± 0.2446, n = 13; T_S MPEPpAkt/Akt: 0.7443 ± 0.1597, n = 8. T_pAkt/Akt versus T_S VehpAkt/Akt, Dunn’s test, *p = 0.044; T_pAkt/Akt versus T_S MPEPpAkt/Akt, Dunn’s test, p > 0.99; T_S VehpAkt/Akt versus T_S MPEPpAkt/Akt, Dunn’s test, *p = 0.011). (D) Schematic of short- and overtraining followed by the post-training omission or devaluation procedures. (E) Averaged time courses of magazine entry (ME) rates (left) and inactive nose-poke (INP) rates (right) during training (Sh, n = 19; Ov, n = 16; ME/min, session: F8,264 = 15, ****p < 0.0001, group: F1,33 = 0 [file pbio.3003288.s001.pdf]

S2 Fig

A

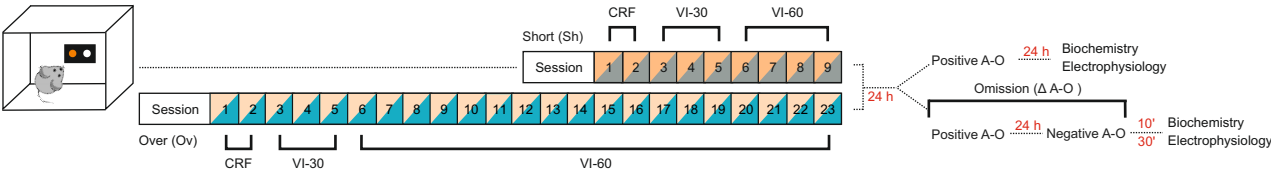

B

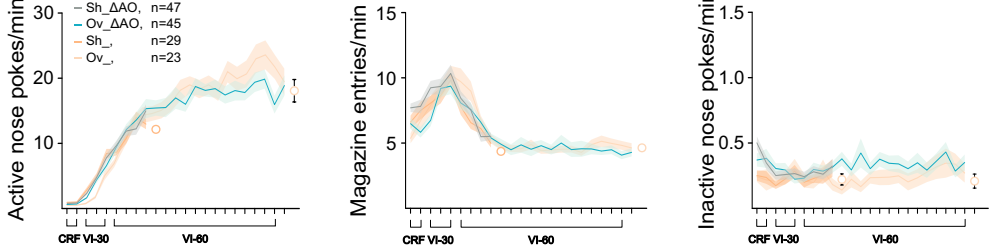

C

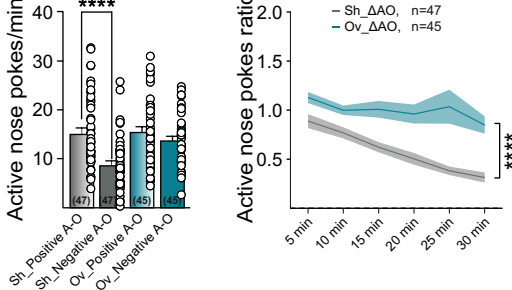

D

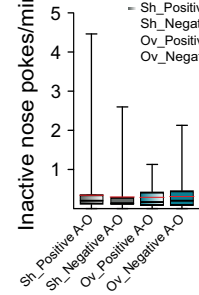

E

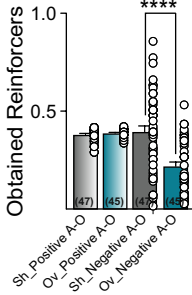

F

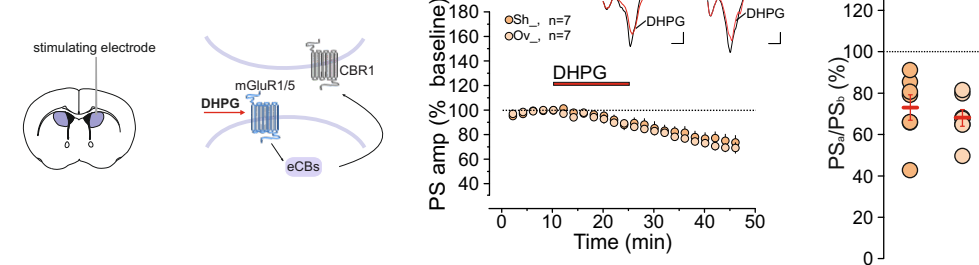

G

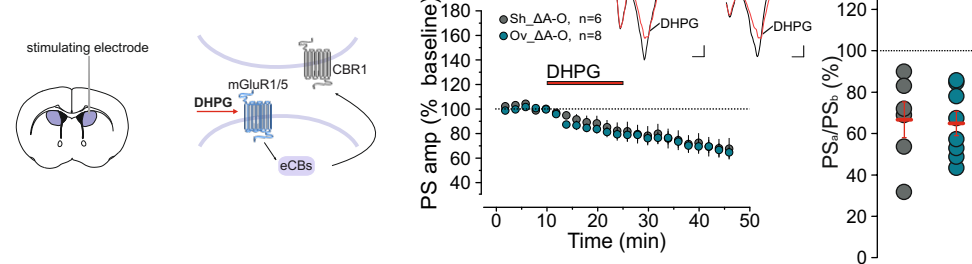

Supplement: S2 Fig — (A) Schematic of behavioral training paradigms. (B) ANP/min (session: F8,1,120 = 301.4, ****p < 0.0001; group: F3,140 = 1.353, p = 0.2; interaction: F24,1,120 = 1.735, *p = 0.015), ME (session: F8,1,120 = 39.06, ****p < 0.0001; group: F3,140 = 1.121, p = 0.34; interaction, F24,1,120 = 2.414, ***p = 0.0002) and INP/min (session: F8,1,120 = 2.649, **p = 0.007; group:: F3,140 = 1.517, p = 0.21; F24,1,120 = 1.416, p = 0.09) during instrumental learning in the different experimental groups (Sh, n = 29; Sh ΔAO, n = 47; Ov, n = 23; Ov ΔAO, n = 45). Symbols represent the positive A–O performance for mice not undergoing omission procedure. (C) Post-training omission procedure. (Left) Comparison of ANP rates between positive and negative A–O contingency in both short- and overtrained mice (A–O contingency: F1,90 = 58.45, ****p < 0.0001, group: F1,90 = 4.837, **p = 0.0304, A–O contingency × group interaction: F1,90 = 21.33, ****p < 0.0001; Sh n = 47, positive A–O: 15.33 ± 1.05, negative A–O: 8.35 ± 0.80, Sidak ****p < 0.0001; Ov n = 45, positive A–O: 15.50 ± 1.002, negative A–O 13.90 ± 0.79; Sidak p = 0.07). (Right) Time courses of ANP ratio that indicate a main group effect (F5,450 = 11.11, ****p < 0.0001; F1,90 = 27.02, ****p < 0.0001; interaction: F5,450 = 3.457, **p = 0.0045. (D–E) Comparison of INP rates (D) and obtained reinforcers (E) between positive and negative A–O contingency, in both short- (n = 47) and overtrained (n = 45) mice (INP/min; A–O contingency: F1,90 = 0.119, p = 0.73, group: F1,90 = 0.047, p = 0.82, A–O contingency × group interaction: F1,90 = 0.84, p = 0.36; Sh, positive A–O: 0.34 ± 0.09, negative A–O: 0.30 ± 0.07, Sidak: p = 0.60; Ov, positive A–O: 0.29 ± 0.045, negative A–O 0.31 ± 0.06; Sidak: p = 0.90; Reinforcers, A–O contingency: F1,90 = 14.7, ***p = 0.0002, group: F1,90 = 25.21, ****p < 0.0001, A–O contingency × group interaction:, F1,90 = 20.9, ****p < 0.0001; Sh_Positive A–O: 0.38 ± 0.006, Ov_Positive A–O: 0.39 ± 0.003; Sidak p = 0.83; Sh_Negat [file pbio.3003288.s002.pdf]

S3 Fig  
A

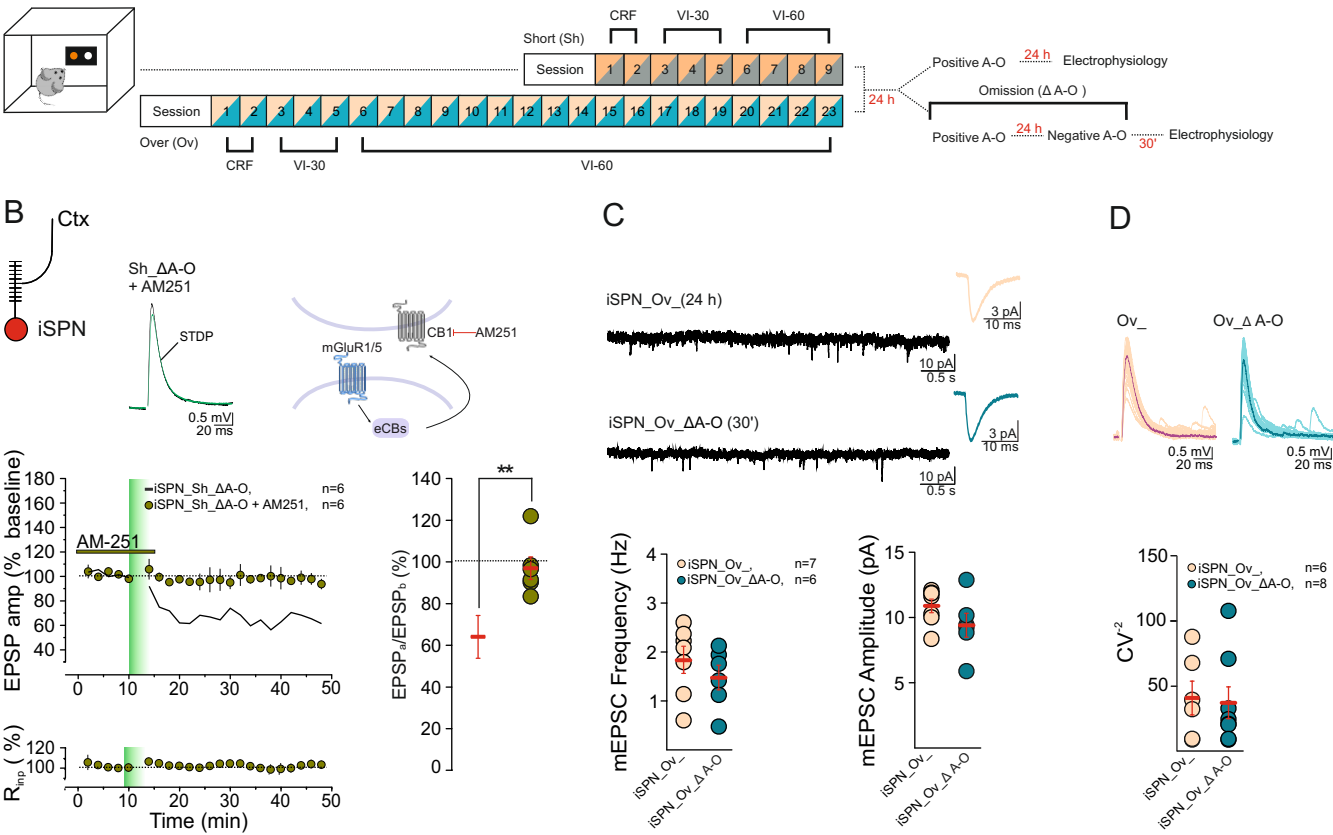

Supplement: S3 Fig — (A) Schematic of the behavioral regimes followed by ex vivo electrophysiology. (B) The CB1 antagonist AM251 (4 μM) prevented t-LTD at cortico-iSPN synapses in Sh_Δ A–O mice (cells n = 6, mice n = 3; F5,22 = 0.4, p = 0.7; iSPN_Sh_Δ A–O versus iSPN_Sh_Δ A–O + AM251, Mann–Whitney test, **p = 0.004, U = 1). Solid black line (average) is the time course from Fig 3C, reported here for comparison. Data are presented as time courses (mean ± SEM) of normalized EPSP amplitudes and normalized Rinp. Scatterplot summarizes the ratios of synaptic responses after (a) and before (b) the STDP. Insets represent superimposed averaged recordings (10 traces) before (black line) and after (green line) the delivery of the STDP protocol (green vertical bar), and the proposed signaling elements targeted by the defined antagonist. (C) (Top) Representative traces for mEPSC recorded at iSPN synapses in Ov_ (24 h later) compared to Ov_Δ Α–Ο (30 min later) mice. (Bottom) Scatterplot comparing mEPSC frequency (Hz) and amplitude (pA) in the two mouse groups (iSPN_Ov_: cells n = 7, mice = 4; frequency 1.82 ± 0.27 Hz, amplitude 10.85 ± 0.5 pA; iSPN_Ov_Δ A–O: cells n = 6, mice = 4; frequency (Hz) 1.46 ± 0.24 Hz, amplitude 9.39 ± 0.90 pA; iSPN_ Ov_ versus iSPN_Ov_Δ A–O: frequency, Mann–Whitney test, p = 0.23, U = 12; amplitude: Mann–Whitney test, p = 0.2, U = 12). (D) (I) Comparison of CV−2 of evoked EPSPs in iSPN_Ov_(cells n = 6, mice n = 5, 41.01 ± 12.98) and iSPN_Ov_Δ A–O (cells n = 8, mice n = 7, 37.33 ± 12.20, iSPN_ Ov_ versus iSPN_Ov_Δ A–O, Mann–Whitney test, p > 0.99, U = 24). CV−2 calculations were based on 60 sweeps (10 min recordings). Data are presented as mean ± SEM. Data set are available at the following link: https://doi.org/10.48557/VCAWUD. (PDF) [file pbio.3003288.s003.pdf]

S4 Fig

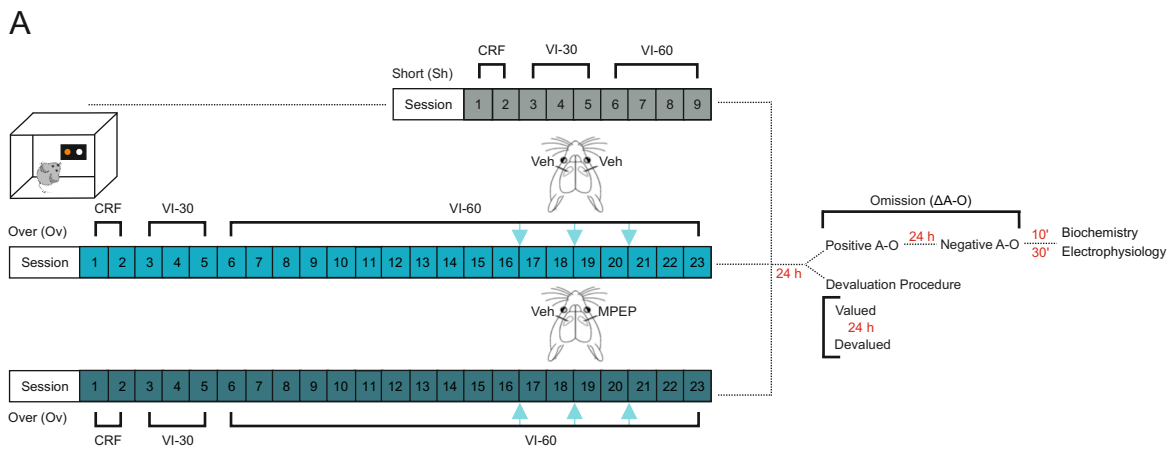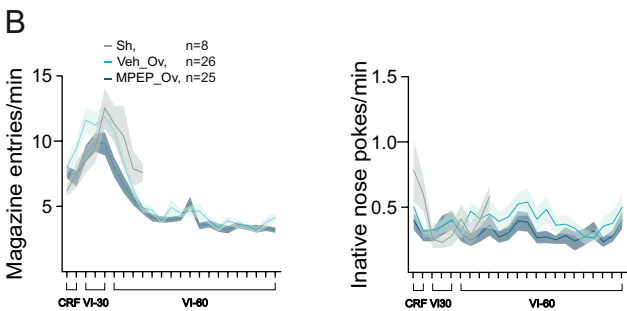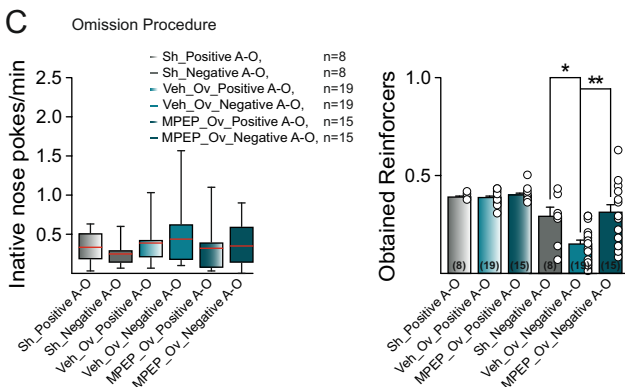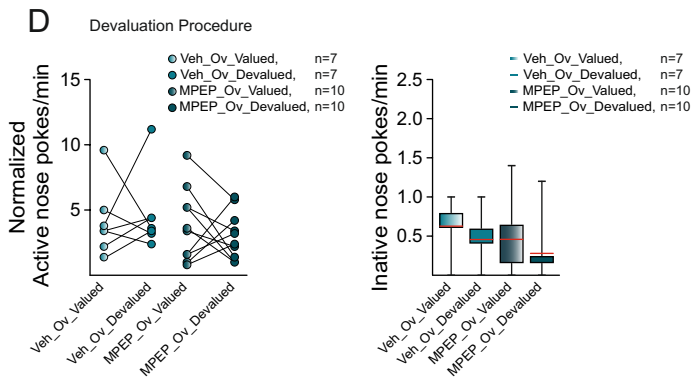

Supplement: S4 Fig — (A) Schematic of the behavioral paradigms and in vivo pharmacological manipulation. (B) ME rates (left) and INP rates (right) during training in Veh_Ov, MPEP_Ov, and in the control Sh group (Sh, n = 8, Veh_Ov, n = 26, MPEP_Ov, n = 25; ME/min, session: F8,448 = 22, ****p < 0.0001; group: F2,56 = 3, p = 0.08; interaction: F8,448 = 3, ***p = 0.0005; INP/min, session: F8,448 = 4, ***p = 0.0004; group: F2,56 = 1.2, p = 0.3; interaction: F8,448 = 1.6, p = 0.06). (C) Comparison of INP rates (left) and obtained reinforcers (right) between positive and negative A–O contingency in the different experimental groups (Sh, n = 8, Veh_Ov, n = 19, MPEP_Ov, n = 15; INP/min; A–O contingency: F1,39 = 0.001, p = 0.97; group: F2,39 = 0.85, p = 0.43; A–O contingency × group interaction, F2,39 = 0.45, p = 0.64; Sh, positive A–O: 0.33 ± 0.07, negative A–O: 0.25 ± 0.6, Sidak: p = 0.87; Veh_Ov, positive A–O: 0.39 ± 0.06, negative A–O: 0.44 ± 0.08, Sidak: p = 0.89; MPEP_Ov, positive A–O: 0.32 ± 0.9, negative A–O: 0.35 ± 07, Sidak: p = 0.98; reinforcers; Sh_Positive A–O: 0.39 ± 0.006, Veh_Ov_Positive A–O: 0.39 ± 0.008; MPEP_Ov_Positive: 0.40 ± 0.009; Sh_Positive A–O versus Veh_Ov_Positive A–O, Sidak p > 0.99, Sh_Positive A–O versus MPEP_Ov_Positive A–O, Sidak p = 0.98, Veh_Ov_Positive A–O versus MPEP_Ov_Positive A–O, Sidak p = 0.96; Sh_Negative A–O, 0.29 ± 0.05, Veh_Ov_Negative A–O, 0.15 ± 0.02, MPEP_Ov_Negative A–O, 0.31 ± 0.04; Sh_Negative A–O versus Veh_Ov_Negative A–O, Sidak ***p = 0.0008, Sh_Negative A–O versus MPEP_Ov_Negative A–O, Sidak p = 0.93,Veh_Ov_Negative A–O versus MPEP_Ov_Negative A–O, Sidak, ****p < 0.0001). (D) Post-training devaluation procedure in the vehicle and MPEP-treated overtrained mice (Veh_Ov, n = 7, MPEP_Ov, n = 10). (Left) ANP rates (Veh_Ov_Valued, 4.11 ± 1.01, Veh_Ov_Devalued, 4.70 ± 1.12, MPEP_Ov_Valued, 3.8 ± 0.87, MPEP_Ov_Devalued, 3.06 ± 0.58; Veh_Ov_Valued versus Veh_Ov_Devalued, Sidak p = 0.91, MPEP_Ov_Valued versus MPEP_Ov_Devalued; Sidak p = 0.76). (Middle [file pbio.3003288.s004.pdf]

S5 Fig

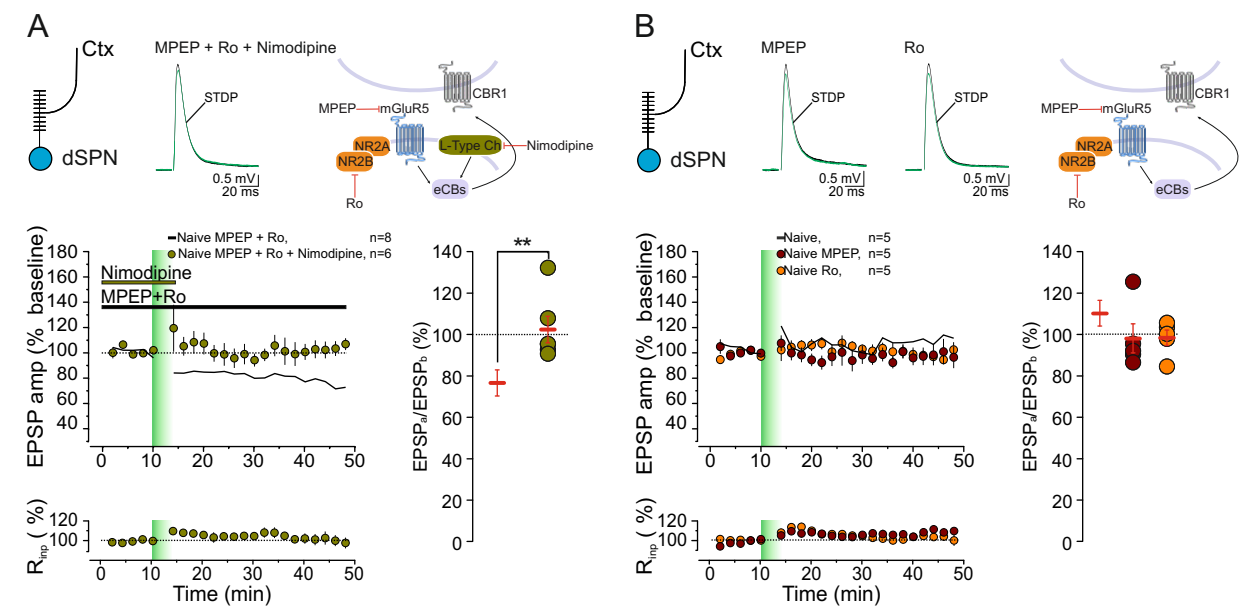

Supplement: S5 Fig — Fig 5. (A) The L-type VGCC blocker nimodipine (10 μM) blocked dSPN t-LTD gated upon co-application of MPEP and Ro-256981 (Ro) during the negative STDP (dSPN_Naïve + MPEP + Ro + Nimodipine, cells n = 6, mice n = 6, F5,22 = 1, p = 0.4; dSPN_Naïve + MPEP + Ro versus dSPN_Naïve + MPEP + Ro + Nimodipine, Mann–Whitney test, **p = 0.008, U = 4). Solid black line (average) is the time course from Fig 5A, reported here for comparison. (B) Application of either MPEP or Ro alone during negative STDP failed to induce t-LTD in dSPNs (dSPN_Naïve + MPEP, cells n = 5, mice n = 4; F4,22 = 0.6, p = 0.6; dSPN_Naïve + Ro, cells n = 5, mice n = 4; F4,22 = 0.97, p = 0.4; group comparison; F2,12 = 1.3, p = 0.3). (A–B) Solid black lines (average) are the time course of MPEP + Ro (A) and control (B) conditions from Fig 5A, reported here for comparison. Data are time courses (mean ± SEM) of normalized EPSP amplitudes and normalized Rinp. Scatterplots are ratios of synaptic responses after (a) and before (b) the STDP. Insets represent superimposed averaged recordings (10 traces) before (black line) and after (green line) the delivery of the STDP protocol (green vertical bar), and the proposed signaling elements targeted by the defined drugs. Data set are available at the following link: https://doi.org/10.48557/VCAWUD. (PDF) [file pbio.3003288.s005.pdf]
