# Supplementary material for: Behavioral inflexibility through overtraining is mediated by reduced mGluR1/5 signaling capacity in the dorsolateral striatum
Source: PLoS Biol. 2025 Jul 29;23(7):e3003288. doi: 10.1371/journal.pbio.3003288 (PMC12327642; doi:10.1371/journal.pbio.3003288)
Supplement: S1 Raw Images — (PDF) [file pbio.3003288.s007.pdf]

S1_raw_images. Original blot images for 1C, 1F, 2B, 2C, 2E, 2F, 2H, 4D, 5D, 5F, S1C

Fig 1C

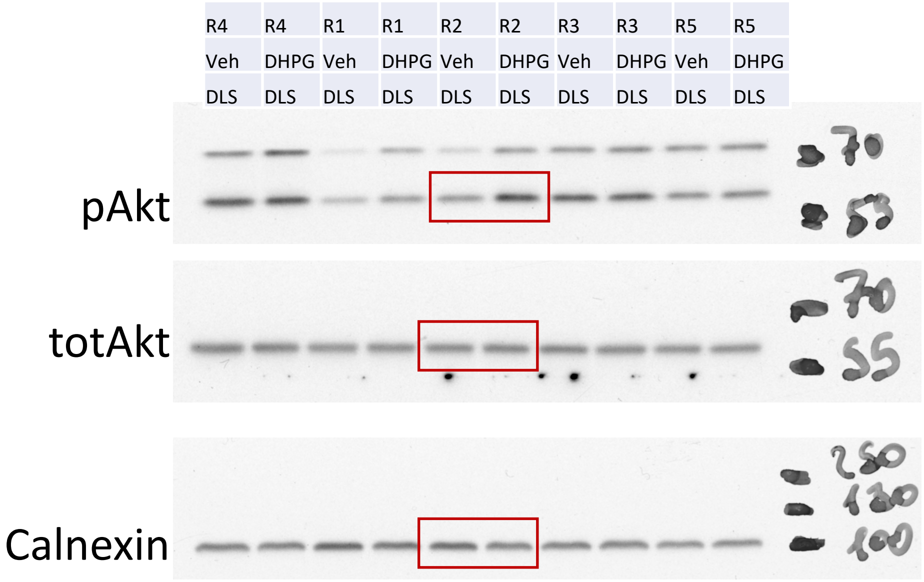
R4 Veh DLS
R4 DHPG DLS
R1 Veh DLS
R1 DHPG DLS
R2 Veh DLS
R2 DHPG DLS
R3 Veh DLS
R3 DHPG DLS
R5 Veh DLS
R5 DHPG DLS
pAkt
totAkt
Calnexin
70
55
70
55
250
130
100

Fig 1F

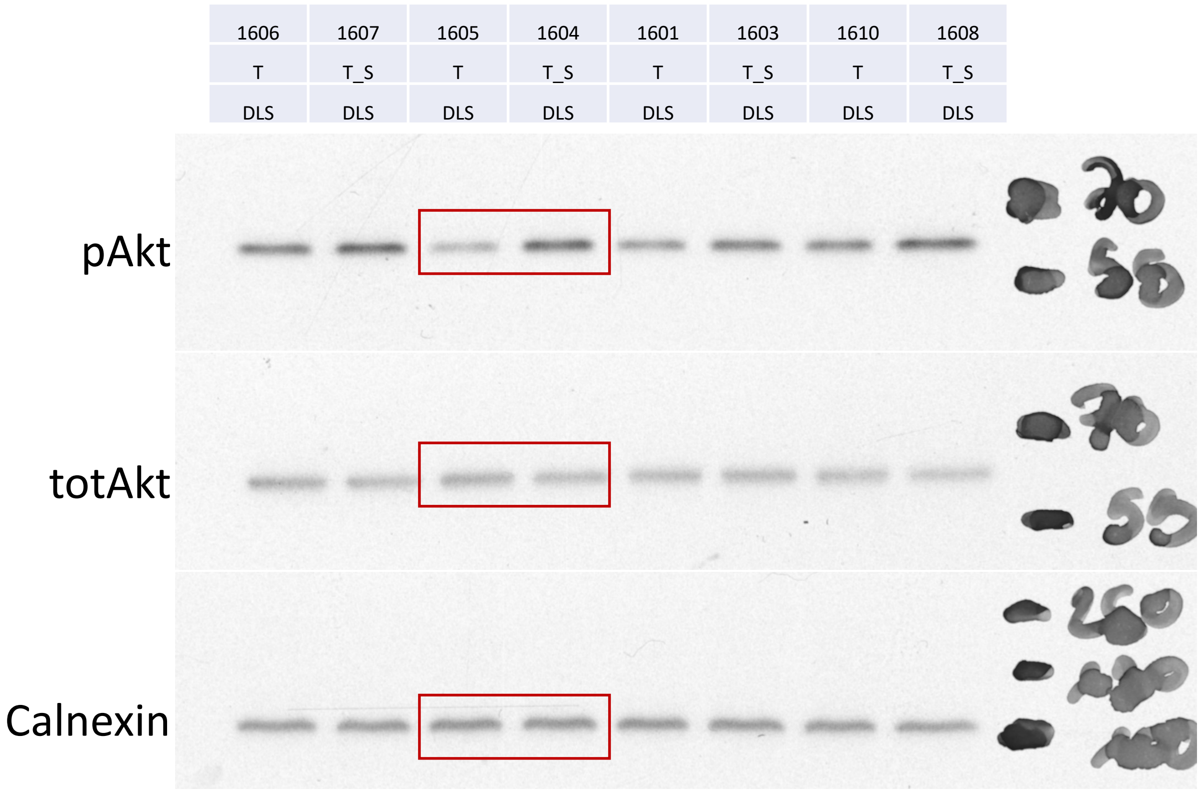

1606
1607
1605
1604
1601
1603
1610
1608
T
T_S
T
T_S
T
T_S
T
T_S
DLS
DLS
DLS
DLS
DLS
DLS
DLS
DLS
pAkt
totAkt
Calnexin

Fig 2B

60729	1279	60729	1279	60730	1280	60730	1280	60721	1282	60721	1282
DMS	DMS	DLS	DLS	DMS	DMS	DLS	DLS	DMS	DMS	DLS	DLS
Sh_	Ov_	Sh_	Ov_	Sh_	Ov_	Sh_	Ov_	Sh_	Ov_	Sh_	Ov_

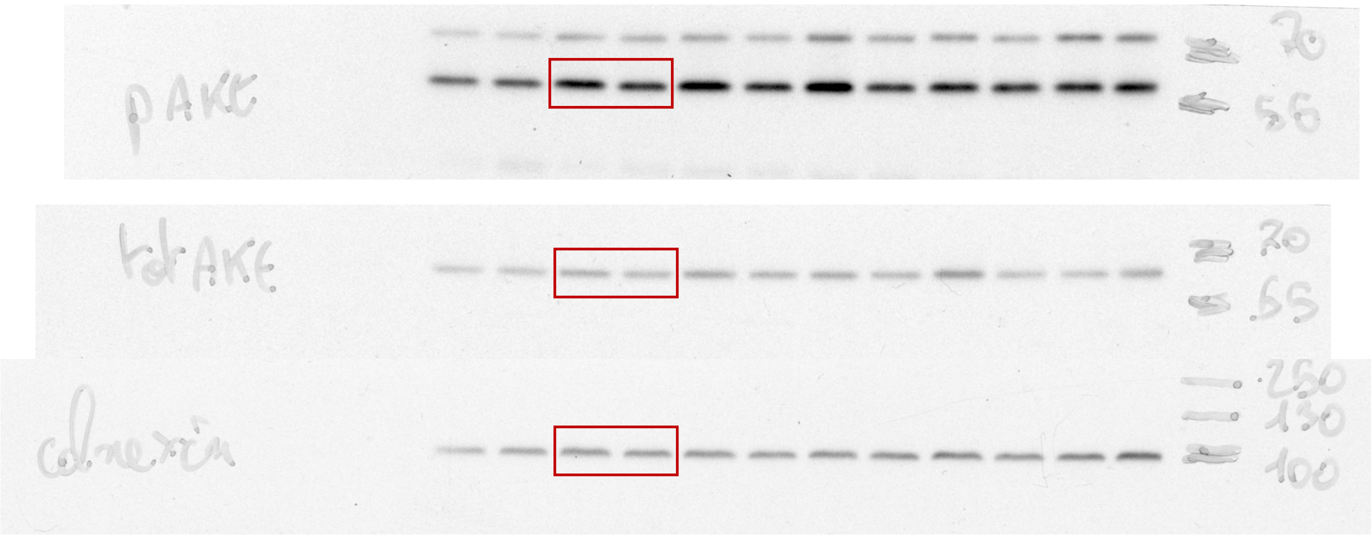

Fig 2C

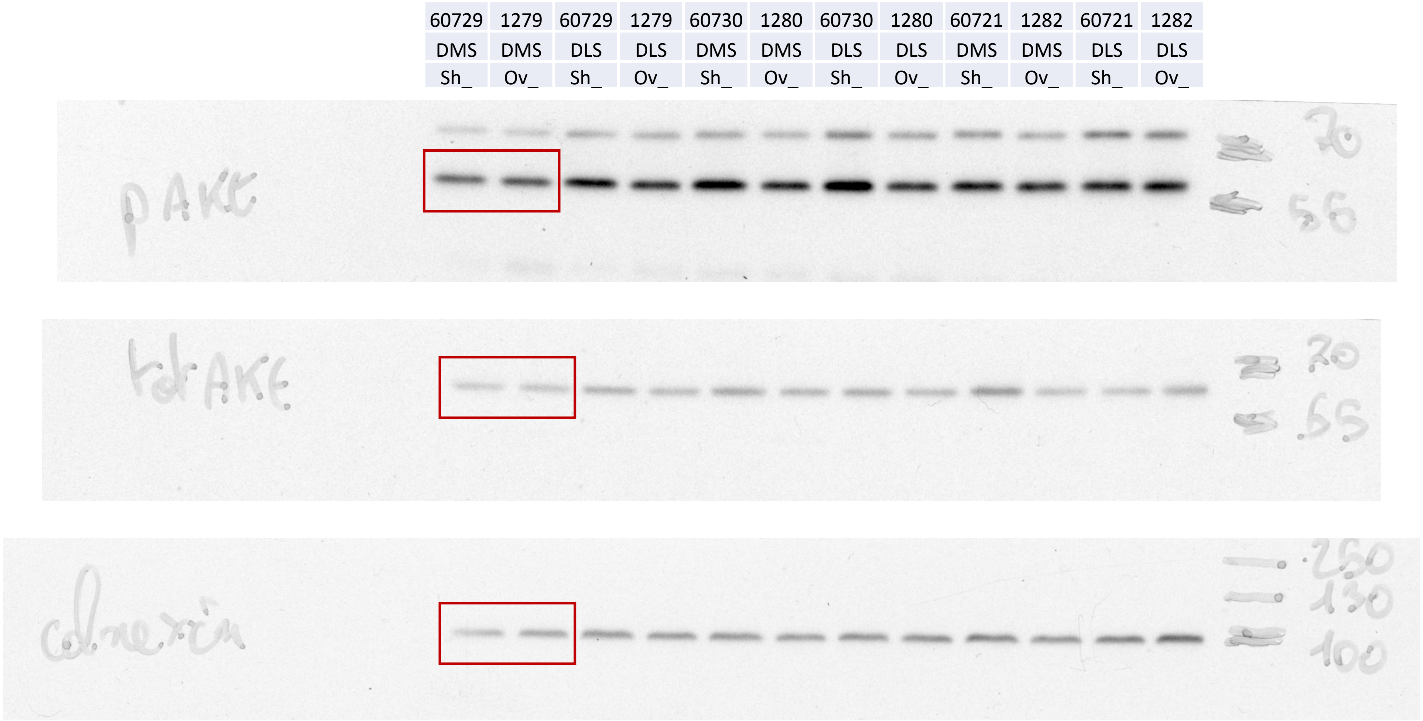
60729 1279 60729 1279 60730 1280 60730 1280 60721 1282 60721 1282
DMS DMS DLS DLS DMS DMS DLS DLS DMS DMS DLS DLS
Sh_ Ov_ Sh_ Ov_ Sh_ Ov_ Sh_ Ov_ Sh_ Ov_ Sh_ Ov_
pAKt
70
56
70
65
calnexin
250
130
100

Fig 2E

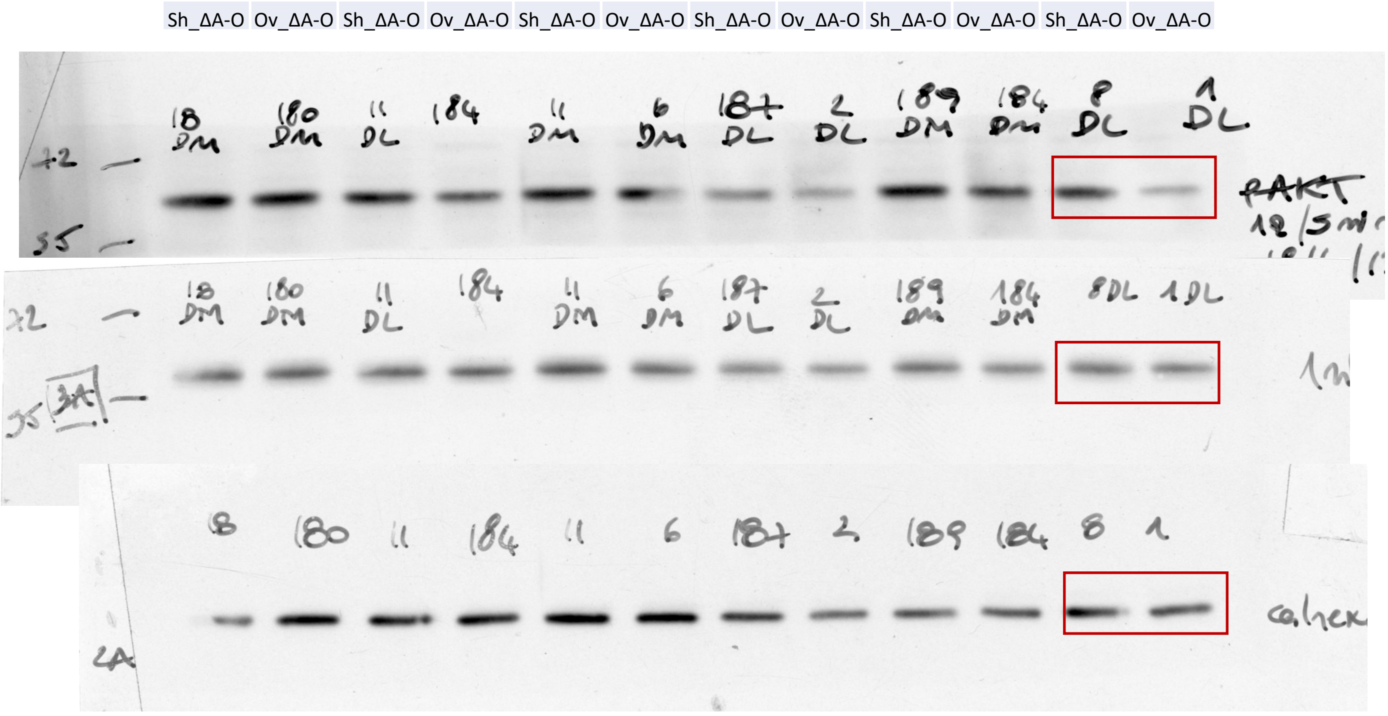
Sh_ΔA-O
Ov_ΔA-O
Sh_ΔA-O
Ov_ΔA-O
Sh_ΔA-O
Ov_ΔA-O
Sh_ΔA-O
Ov_ΔA-O
Sh_ΔA-O
Ov_ΔA-O
Sh_ΔA-O
Ov_ΔA-O

Fig 2F

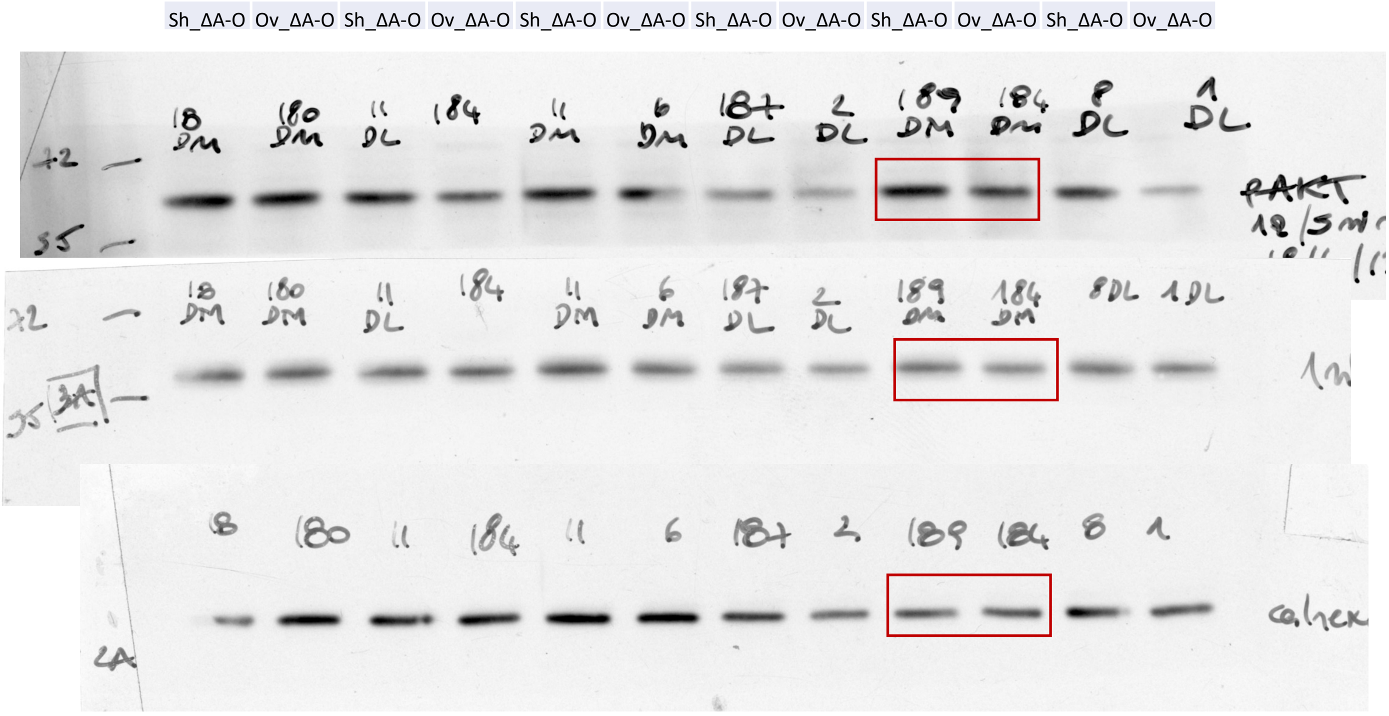
Sh_ΔA-O
Ov_ΔA-O
Sh_ΔA-O
Ov_ΔA-O
Sh_ΔA-O
Ov_ΔA-O
Sh_ΔA-O
Ov_ΔA-O
Sh_ΔA-O
Ov_ΔA-O
Sh_ΔA-O
Ov_ΔA-O

Fig 2H

60728	1189	1287	1167		1279	1164	1282	1163
Sh_	Sh_ΔA-O	Sh_	Sh_ΔA-O		Ov_	Ov_ΔA-O	Ov_	Ov_ΔA-O
DLS	DLS	DLS	DLS		DLS	DLS	DLS	DLS

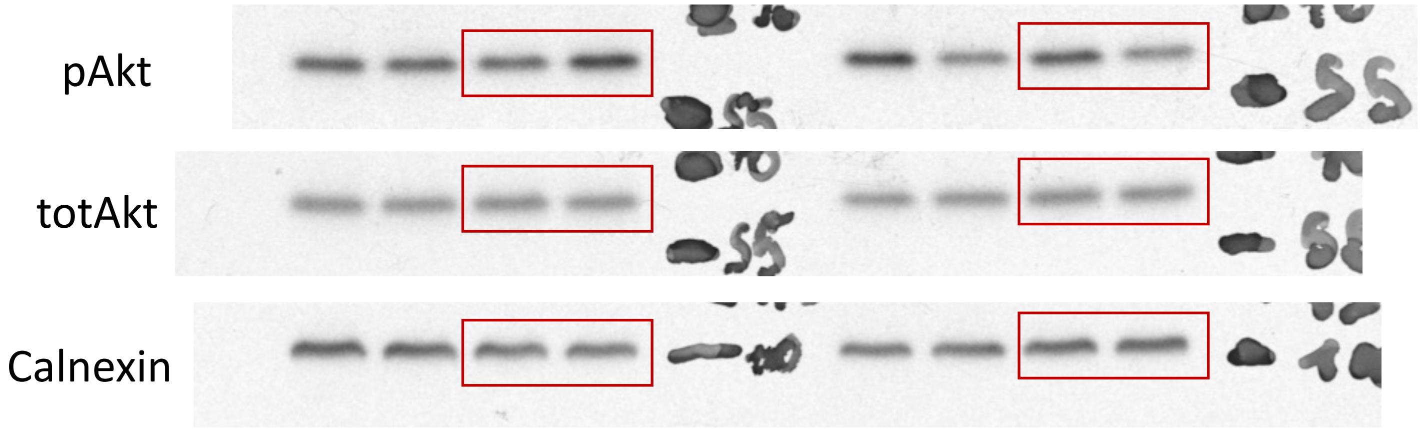

Fig 4D

2110	1507	2210	2106	2104	2128	2109	2101	2131
-	Veh	MPEP	-	Veh	MPEP	-	Veh	MPEP
Sh_Δ A-O	Ov_Δ A-O	Ov_Δ A-O	Sh_Δ A-O	Ov_Δ A-O	Ov_Δ A-O	Sh_Δ A-O	Ov_Δ A-O	Ov_Δ A-O
DLS	DLS	DLS	DLS	DLS	DLS	DLS	DLS	DLS

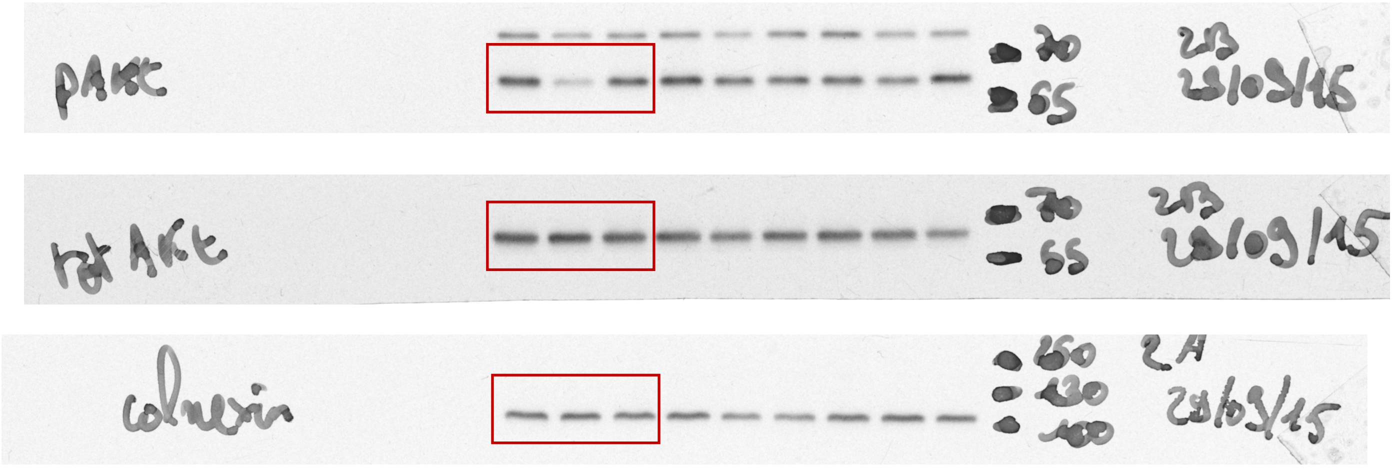

Fig 5D

60723	1175	1282	1064	60730	1170	1280	1065	60731	1189	1281	1173
DLS	DLS	DLS	DLS	DLS	DLS	DLS	DLS	DLS	DLS	DLS	DLS
Sh_	Sh_ΔA-O	Ov_	Ov_ΔA-O	Sh_	Sh_ΔA-O	Ov_	Ov_ΔA-O	Sh_	Sh_ΔA-O	Ov_	Ov_ΔA-O

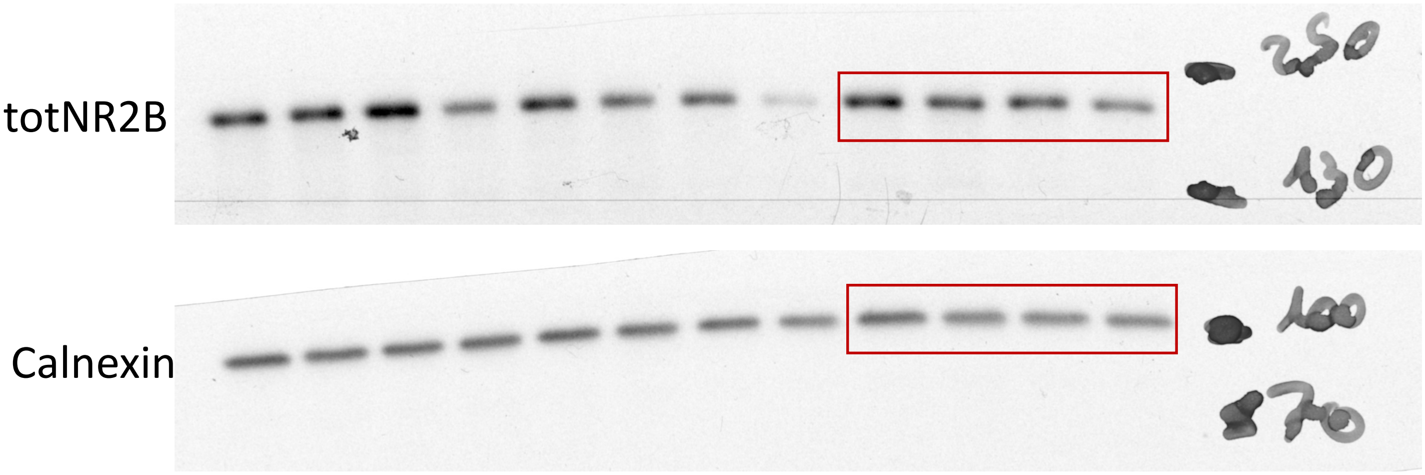

Fig 5F

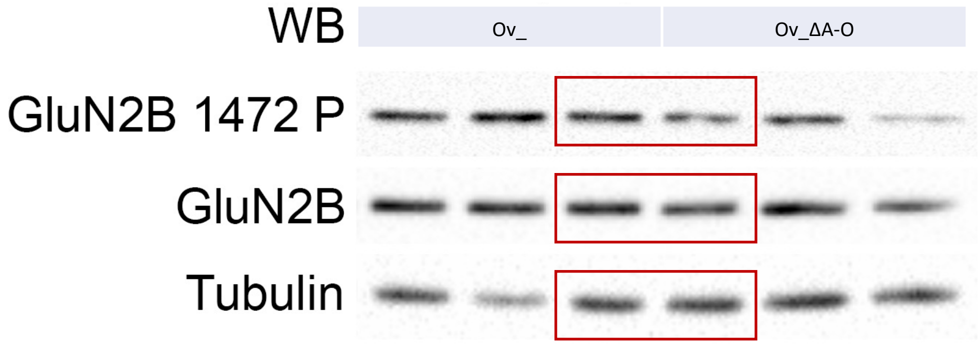

Fig S1C

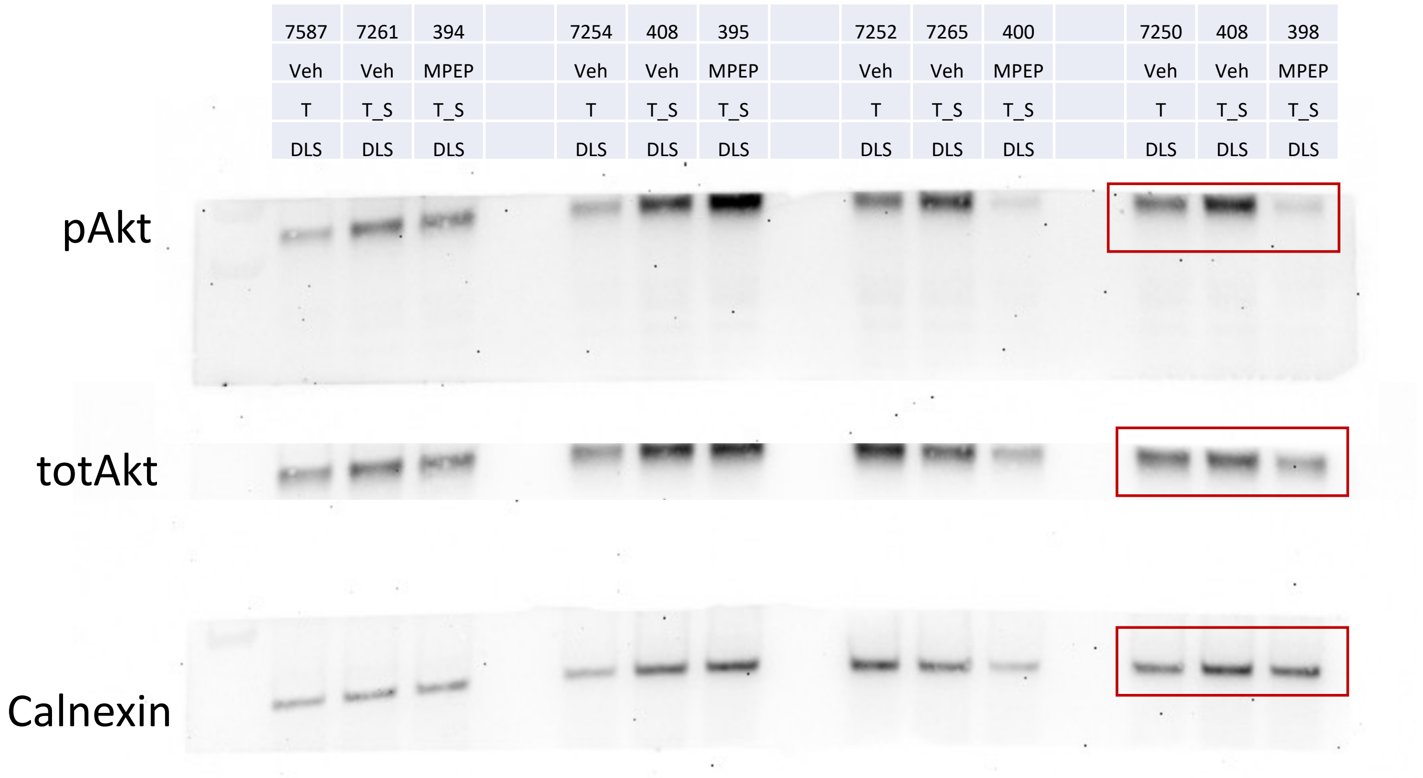
7587 7261 394 7254 408 395 7252 7265 400 7250 408 398
Veh Veh MPEP Veh Veh MPEP Veh Veh MPEP Veh Veh MPEP
T T_S T_S T T_S T_S T T_S T_S T T_S T_S
DLS DLS DLS DLS DLS DLS DLS DLS DLS DLS DLS DLS
pAkt
totAkt
Calnexin
